# Optimal timing and effectiveness of COVID-19 outbreak responses in China: a modelling study

**DOI:** 10.1186/s12889-022-12659-2

**Published:** 2022-04-07

**Authors:** Anthony Zhenhuan Zhang, Eva A. Enns

**Affiliations:** 1 Department of Industrial and Systems Engineering, University of Minnesota, 100 Union St SE, Minneapolis, 55455 USA; 2 Division of Health Policy and Management, University of Minnesota School of Public Health, 420 Delaware St SE, MMC 729 Mayo, Minneapolis, 55455 USA

**Keywords:** COVID-19, Public health interventions, Economic burden, Health burden

## Abstract

**Background:**

In January 2020, an outbreak of atypical pneumonia caused by a novel coronavirus, SARS-CoV-2, was reported in Wuhan, China. On Jan 23, 2020, the Chinese government instituted mitigation strategies to control spread. Most modeling studies have focused on projecting epidemiological outcomes throughout the pandemic. However, the impact and optimal timing of different mitigation approaches have not been well-studied.

**Methods:**

We developed a mathematical model reflecting SARS-CoV-2 transmission dynamics in an age-stratified population. The model simulates health and economic outcomes from Dec 1, 2019 through Mar 31, 2020 for cities including Wuhan, Chongqing, Beijing, and Shanghai in China. We considered differences in timing and duration of three mitigation strategies in the early phase of the epidemic: city-wide quarantine on Wuhan, travel history screening and isolation of travelers from Wuhan to other Chinese cities, and general social distancing.

**Results:**

Our model estimated that implementing all three mitigation strategies one week earlier would have averted 35% of deaths in Wuhan (50% in other cities) with a 7% increase in economic impacts (16-18% in other cities). One week’s delay in mitigation strategy initiation was estimated to decrease economic cost by the same amount, but with 35% more deaths in Wuhan and more than 80% more deaths in the other cities. Of the three mitigation approaches, infections and deaths increased most rapidly if initiation of social distancing was delayed. Furthermore, social distancing of working-age adults was most critical to reducing COVID-19 outcomes versus social distancing among children and/or the elderly.

**Conclusions:**

Optimizing the timing of epidemic mitigation strategies is paramount and involves weighing trade-offs between preventing infections and deaths and incurring immense economic impacts. City-wide quarantine was not as effective as city-wide social distancing due to its much higher daily cost than social distancing. Under typical economic evaluation standards, the optimal timing for the full set of control measures would have been much later than Jan 23, 2020 (status quo).

**Supplementary Information:**

The online version contains supplementary material available at (10.1186/s12889-022-12659-2).

## Background

In January 2020, an outbreak of atypical pneumonia caused by a novel coronavirus, SARS-CoV-2, was reported in Wuhan, China. The outbreak quickly grew and expanded to other cities and countries. At the end of January, there were nearly 10,000 confirmed COVID-19 cases, and over 200 reported deaths in China [1]. On Jan 23, 2020, the Chinese government instituted a variety of mitigation strategies to control viral spread, including a city-wide quarantine in the city of Wuhan, mass social distancing in other Chinese cities, and identification and isolation of those in other major Chinese cities with recent travel to Wuhan [2]. On Jan 31, 2020, the WHO recognized the COVID-19 outbreak as a “Public Health Emergency of International Concern”; on Mar 11, 2020, COVID-19 was declared a global pandemic [1].

Despite the actions taken by the Chinese government, the number of confirmed COVID-19 cases grew to 45,000 in mid-February and 80,000 by early March [1]. Initially, the Chinese government was criticized for not acting quickly enough to contain the outbreak. However, as global spread continued and other regions struggled to address sustained local transmission, countries like China have been praised for their swift enactment of extensive control measures [3, 4]. By April, new cases in China were declining, while other regions were still experiencing initial waves of exponential growth [1].

In responding to an infectious disease outbreak, governments are faced with questions of both when to enact control measures, such as mandated social distancing or city-wide quarantining, and for how long. Enacting measures early stems transmission and results in fewer cases and deaths; however, extreme measures come at substantial economic costs and disruption to daily life [5, 6]. On the other hand, actions that come too late will have allowed more widespread transmission that may ultimately be more costly to contain.

Previous studies evaluating COVID-19 control measures have primarily focused on epidemiological outcomes only, with no consideration of economic impacts. For example, Wu et. al. [7] conducted a simulation study using a susceptible-exposed-infectious-recovered (SEIR) metapopulation model to simulate SARS-CoV-2 transmission and estimate the extent of domestic and international spread. Leung et. al. [8] used a similar model to investigate the impact of relaxing control measures on COVID-19 cases after the first wave of infection in China. Prem et. al. [9] used an age-structured SEIR model to assess the impact of different social mixing patterns on COVID-19 epidemiological outcomes in Wuhan. None of the aforementioned studies have studied the trade-off between averting COVID-19 through social and economic restrictions and the economic costs of these policies.

In this study, we use a mathematical model of COVID-19 to explore how differences in the timing and duration of mitigation strategies would have impacted both epidemiological and economic outcomes in Wuhan, China, the epicenter of the epidemic, as well as in three highly-connected Chinese cities (Chongqing, Beijing, and Shanghai). While our model specifically focuses on the situation in these four Chinese cities, our analysis provides insights into decision-making trade-offs in the context of the COVID-19 epidemic mitigation that are applicable to other localities.

## Methods

We developed a mathematical model to reflect SARS-CoV-2 transmission dynamics within and between four major Chinese cities: Wuhan, Chongqing, Beijing, and Shanghai. We modeled the outbreak as starting on Dec 1, 2019 with a zoonotic source in Wuhan. While there is some evidence that SARS-CoV-2 may have been circulating at low levels 1-2 months prior, sustained viral transmission was likely established in late November or early December, justifying our start date [10]. We simulated outcomes through Mar 31, 2020 to capture the early health and economic impacts of the SARS-CoV-2 pandemic and mitigation strategies reflected in the first quarter of 2020. We calibrated the model to match observed COVID-19 morbidity and mortality statistics over the simulation period in Wuhan [11, 12], accounting for the actual timing of control measures put in place by the Chinese government as the outbreak evolved. We then used the calibrated model to predict epidemiological and economic outcomes over the simulated time horizon under the status quo and counterfactual policy mitigation scenarios.

**Fig. 1:**
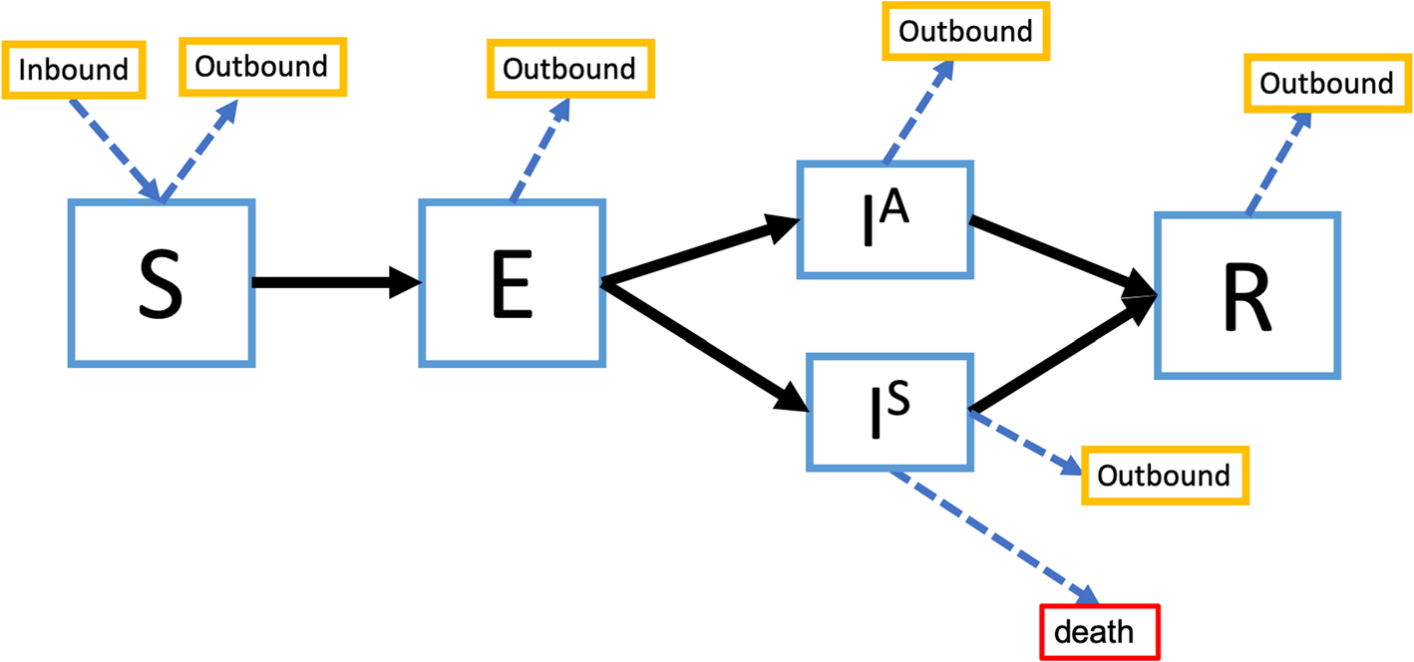
State transition diagram for the Wuhan model. States include Susceptible (*S*), Exposed (*E*), Infected and asymptomatic (*I*^*A*^), Infected and symptomatic (*I*^*S*^), and Recovered (*R*). Each day, individuals may stay in their current health state, progress to another possible health state, leave Wuhan (outbound travel), or arrive in Wuhan (inbound travel). We assumed that individuals arriving in Wuhan were all susceptible. Given the short time horizon, we only modeled death due to symptomatic COVID-19 infections. All compartments were stratified by age

**Fig. 2:**
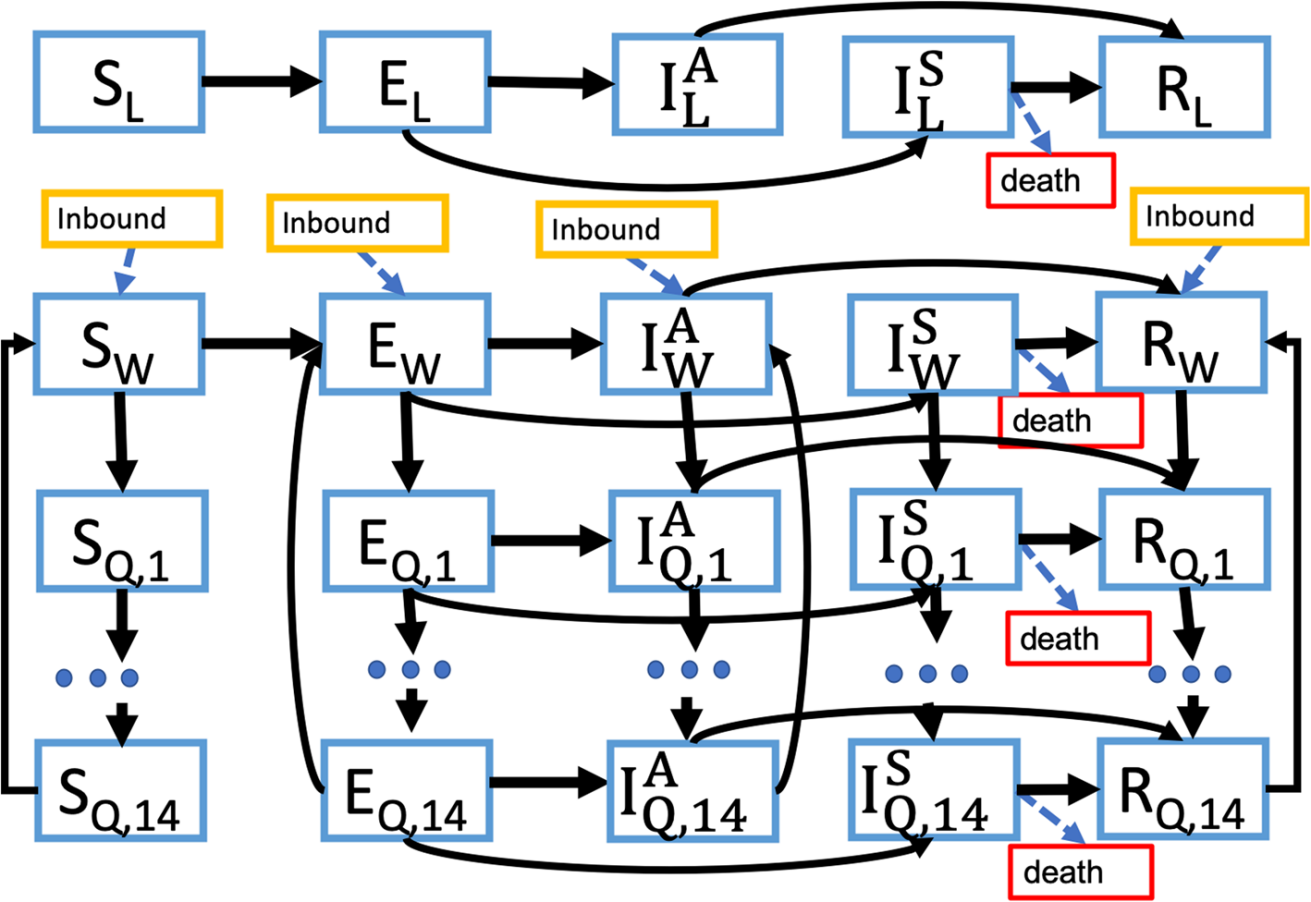
State transition diagram for models of other Chinese cities (Chongqing, Beijing, and Shanghai). States include Susceptible (*S*), Exposed (*E*), Infected and asymptomatic (*I*^*A*^), Infected and symptomatic (*I*^*S*^), and Recovered (*R*). Subscript *L* denotes compartments for the local city population, while subscript *W* denotes individuals who have arrived from Wuhan. Compartments with subscripts *Q*_1_ through *Q*_14_ are used to represent a 14-day isolation period for travelers arriving from Wuhan. When travel history screening is active, individuals arriving from Wuhan enter the set of *Q*_1_ compartments. After progressing through the 14 quarantine states, individuals return to the corresponding non-quarantined compartments, except for those in the *I*^*S*^ compartment who remain quarantined until their illness is resolved (either through recovery or death)

We present the health state transition diagrams in Figs. 1 and 2. Model parameters are summarized in Table 1. Additional model details, including the differential equations governing the model dynamics, are provided in Additional file 1 — Technical Appendix, Section 1. The simulation source code can be found on Github at: https://github.com/Anthony-zh-Zhang/COVID_19_model.

**Table 1:** Model input parameters and data sources

Variable	Description	Value (range)	Source
	**Transmission parameters**		
*β*_*I*_	per-contact SARS-CoV-2 transmission rate	3.90% (3.86% - 3.94%)	Calibration
*z*	Daily zoonotic force of infection before closure	86	Wu et al. [7]
	**COVID-19 daily mortality rate in Wuhan, by age category**		
*μ*_1_	Youth	0.01% (0.00% - 0.01%)	Calibration
*μ*_2_	Adult	0.55% (0.47% - 0.63%)	Calibration
*μ*_3_	Elderly	5.61% (5.34% - 5.86%)	Calibration
	Mortality rate reduction for other cities, relative to Wuhan	85.7*%*	China CDC [12]
	**Time from symptom onset to death, by age category**		
*μ*_1_	Youth	20 days	Wang et al. [13]
*μ*_2_	Adult	20 days	Wang et al. [13]
*μ*_3_	Elderly	11 days	Wang et al. [13]
	Daily number of contacts, by age category		
*M*_1_	Youth	21	Estimated from Read et al. [14]
*M*_2_	Adult	20	Estimated from Read et al. [14]
*M*_3_	Elderly	15	Estimated from Read et al. [14]
	**Assortative mixing coefficient, by age**		
*γ*_1_	Youth	45.7%	Estimated from Read et al. [14]
*γ*_2_	Adult	78.0%	Estimated from Read et al. [14]
*γ*_3_	Elderly	5.5%	Estimated from Read et al. [14]
	**Wuhan age distribution**		
	Youth	18.89%	National Bureau of Statistics [15]
	Adult	68.43%	National Bureau of Statistics [15]
	Elderly	12.68%	National Bureau of Statistics [15]
	**Chongqing age distribution**		
	Youth	24.78%	National Bureau of Statistics [15]
	Adult	57.80%	National Bureau of Statistics [15]
	Elderly	17.42%	National Bureau of Statistics [15]
	**Beijing age distribution**		
	Youth	14.07%	National Bureau of Statistics [15]
	Adult	73.39%	National Bureau of Statistics [15]
	Elderly	12.54%	National Bureau of Statistics [15]
	**Shanghai age distribution**		
	Youth	13.48%	National Bureau of Statistics [15]
	Adult	71.44%	National Bureau of Statistics [15]
	Elderly	15.07%	National Bureau of Statistics [15]
	**Proportion of infections that are symptomatic**		
*ρ*_1_	Youth	30.3% (5.4% - 65.3%)	Calibration
*ρ*_2_	Adult	67.5% (61.8% - 74.3%)	Calibration
*ρ*_3_	Elderly	42.8% (39.0% - 46.5%)	Calibration
	**Proportion of symptomatic infections that receive clinical confirmation**		
	Youth	9.5% (3.0% - 25.8%)	Informed from model
	Adult	81.9% (69.2% - 94.3%)	Informed from model
	Elderly	100%	Informed from model
	**Travel volume in Wuhan (persons per day)**		
*T*^(*w*,*c*)^(*t*)	outbound, normal,	505,646	Wu et.al [7]
*T*^(*w*,*c*)^(*t*)	outbound, *chunyun*,	720,859	Wu et.al [7]
*T*^(*c*,*w*)^(*t*)	inbound, normal,	490,856	Wu et.al [7]
*T*^(*c*,*w*)^(*t*)	inbound, *chunyun*,	814,046	Wu et.al [7]
	inbound reduction during city-wide quarantine	95.62%	Baidu database [16]
	outbound reduction during city-wide quarantine	92.37%	Baidu database [16]
	**Proportion of outbound Wuhan travel volume arriving in other Chinese cities**		
	Chongqing, normal	1.27%	Baidu database [16]
	Beijing, normal	0.86%	Baidu database [16]
	Shanghai, normal	0.66%	Baidu database [16]
	Chongqing, quarantine	0.44%	Baidu database [16]
	Beijing, quarantine	0.24%	Baidu database [16]
	Shanghai, quarantine	0.29%	Baidu database [16]
	**City population size**		
	Wuhan	19 million	Wu et al. [7]
	Chongqing	30.48 million	National Bureau of Statistics [15]
	Beijing	21.54 million	National Bureau of Statistics [15]
	Shanghai	24.24 million	National Bureau of Statistics [15]
	**Daily cost of mass social distancing**		
	Wuhan	$20.41 million USD	Additional file 1 — Technical Appendix, Section 2
	Chongqing	$48.45 million USD	Additional file 1 — Technical Appendix, Section 2
	Beijing	$72.66 million USD	Additional file 1 — Technical Appendix, Section 2
	Shanghai	$78.37 million USD	Additional file 1 — Technical Appendix, Section 2
	Daily cost of Wuhan city-wide quarantine	$522.77 million USD	Additional file 1 — Technical Appendix, Section 2
	Daily cost of contact tracing and quarantine, per person	$16.438 USD	Armbruster and Brandeau [17]
	Health care cost per hospitalized COVID-19 case	$4,124.64 USD	Du et al. [18]
	**Economic cost per non-fatal COVID-19 case**		
	Youth	$395.82 USD	Additional file 1 — Technical Appendix, Section 2
	Adult	$3,951.24 USD	Additional file 1 — Technical Appendix, Section 2
	Elderly	$462.66 USD	Additional file 1 — Technical Appendix, Section 2
	**Economic cost per fatal COVID-19 case**		
	Youth	$2,069,041 USD	Additional file 1 — Technical Appendix, Section 2
	Adult	$1,142,677 USD	Additional file 1 — Technical Appendix, Section 2
	Elderly	$291,130 USD	Additional file 1 — Technical Appendix, Section 2

### Disease transmission and progression

SARS-CoV-2 transmission within each city was modeled following a Susceptible-Exposed-Infected-Recovered (SEIR) dynamic compartmental model, which is commonly used for COVID-19 modelling [7–9]. The modeled population was stratified into three age categories: 0-19 years old (“Youth”), 20-59 years old (“Adults”), and 60 years old and older (“Elderly”). This stratification allowed us to capture age-specific mortality rates, differences in contact patterns, and age-targeted social distancing strategies.

The proportion of susceptible individuals becoming infected at each time step in a given age category was calculated by multiplying the number of contacts of each age category, the age-specific prevalence of SARS-CoV-2 infection at that time step, and the per-contact transmission rate. The contact mixing matrix, which summarizes the number of daily contacts for each age category with every other age category, was estimated from an age-mixing study in Southern China [14].

The daily per-contact transmission rate was estimated through calibration. We assumed that symptomatic and asymptomatic infections had the same per-contact probability of transmission due to similar levels of viral shedding regardless of symptom status [19, 20]. We varied this assumption in the sensitivity analysis. Susceptible individuals who become infected first transition to the exposed compartment. The rates of progression from the exposed to the infected compartment and from the infected to the recovered compartment were calculated to reflect the average incubation and infectious periods, respectively. Age-specific COVID-19 mortality rates were estimated through calibration. In this model, as a simplification, we do not explicitly include separate health care utilization states; however, we do account for health care costs that reflect age-specific rates of health care utilization as well as the delays between infection and death, for fatal COVID-19 cases, reflecting the long hospital stays of severe COVID-19 cases. The infectious period here reflects the time from infectiousness until self-isolation due to symptoms or hospitalization. Given that the duration of hospitalization can be extensive, we included a delay in the aggregation of deaths to account for the time spent hospitalized prior to death. Thus, individuals in the “Youth” and “Adult” age categories who transitioned to death were counted 20 days later to account for this delay, while individuals in the “Elderly” age category had a 11-day reporting delay, given their higher likelihood of rapid decline [13].

### Inter-city travel

The model also included terms reflecting travel to and from Wuhan. Travel volumes were taken to be the same as those estimated by Wu et al. [7]. All incoming travelers to Wuhan were assumed to arrive susceptible over the simulated time horizon given the relatively low prevalence of SARS-CoV-2 infection outside of Wuhan over this time period. The prevalence of infection among outbound travelers from Wuhan on a given day was assumed to be the same as the prevalence within Wuhan. For the cities of Chongqing, Beijing, and Shanghai, the proportion of Wuhan outbound travelers arriving in each city was estimated from a publicly available database reporting daily travel volumes between Chinese cities estimated from location data collected by Baidu, a Chinese internet search engine company [16]. The database provides estimates of travel patterns between Chinese cities from January 1, 2020 onward [16].

### Control measures

The model allowed for three different types of control measures (or mitigation strategies) to mitigate SARS-CoV-2 spread: 1) social distancing (either society-wide or among certain age groups); 2) travel history-based quarantining for those with recent travel to Wuhan (applicable only to Chongqing, Beijing, and Shanghai), and 3) city-wide quarantine (applicable only to Wuhan). Social distancing was assumed to reduce the number of daily contacts by 50% among applicable age categories [21]. Travel history-based quarantining was defined as identifying and isolating individuals with recent travel to Wuhan within a certain time window (14 days in our model). In the base case, we assumed that travel history-based quarantining was 100% effective (varied in sensitivity analysis) at identifying all individuals who had arrived from Wuhan within 14 days of the strategy implementation and accurately identifying and isolated all Wuhan-originating individuals upon arrival in each city. We varied this assumption in the sensitivity analysis. While quarantined, individuals were assumed to have no contact with others, eliminating potential transmission or infection. We only considered a city-wide quarantine for Wuhan, the epicenter of the epidemic, which had the effect of reducing outbound and inbound travel volumes by 92.37% and 95.62%, respectively, as observed in Baidu travel data [16].

To accommodate the potential policy of quarantining of travelers in Chongqing, Beijing, and Shanghai who have recently arrived from Wuhan, the models for these three cities include compartments stratified by travel history (either having recently arrived from Wuhan or having had no recent travel from Wuhan). When the travel-based quarantining policy is inactive, this stratification makes no difference to the model; however, when the policy is activated, a proportion of individuals with travel history from Wuhan are immediately moved to a set of quarantined states where contact is eliminated. Additionally, once the travel history-based quarantining strategy is introduced, a proportion *α* of individuals newly arriving from Wuhan transition directly into the quarantined states. Varying *α* allows us to consider different levels of effectiveness of the travel screening efforts in identifying and reaching recent arrivals. Further details on these calculations are provided in Additional file 1 — Technical Appendix.

### Model calibration and validation

The per-contact SARS-CoV-2 transmission rate and age-specific COVID-19 infection mortality rates were estimated through model calibration. We used Incremental Mixture Importance Sampling (IMIS) [22, 23], a Bayesian calibration approach, to estimate the posterior probability distributions for the daily per-contact transmission rate, the age-specific proportion of asymptomatic infections, and age-specific COVID-19 mortality rates. Calibration targets included clinically confirmed cases in the Elderly Group and cumulative deaths reported in surveillance data through Mar 9, 2020 in Wuhan [11]. We also calibrated to the age-distribution of COVID-19 deaths reported as of Feb 11, 2020 in Wuhan, which was published in a special public health report [12]. Model output on the corresponding date for each target was used to compute a likelihood goodness-of-fit (i.e. Mar 9, 2020 for cumulative Elderly cases and deaths; Feb 11, 2020 for the age-distribution of cumulative deaths). We assumed all “Elderly” individuals with symptomatic infections were diagnosed and confirmed. In cities other than Wuhan, mortality rates were assumed to be 85.7% lower than Wuhan due to a less overwhelmed healthcare infrastructure based on a surveillance report describing the clinical outcomes of COVID-19 patients during the early phase of the epidemic in China [12].

We validated the calibrated model by comparing our simulated model outcome (i.e., cumulative death, cumulative clinical confirmed cases) under the status quo scenario (details below) with the actual epidemiological data in the four cities.

### Status quo and counterfactual scenarios

Our status quo simulations captured the actual timing of control measures as they were announced and implemented. Thus, in the status quo, all three control measures were instituted on Jan 23, 2020 [2]. In the status quo, cities other than Wuhan, workers (in the “Adult” age category) and students (the “Youth” age category) ended their social distancing practices on Feb 29, 2020, and March 31, 2020 respectively [24, 25]. We assumed that the “Elderly” age category continued social distancing practices through the end of the time horizon, given the high risk of COVID-19 morbidity and mortality in that age category. In Wuhan, all control measures continued through the end of our simulation time horizon (Mar 31, 2020), as restrictions were not lifted until April 8, 2020 [26].

We then used the model to simulate counterfactual scenarios by varying the start dates and duration of the different control measures, which we compare to the status quo (the actual timing of measures taken). In one analysis we varied the implementation start date of all three mitigation strategies, while keeping the same end dates as the status quo. In another analysis, we considered different duration of the workplace and school closures in cities other than Wuhan while keeping Wuhan under quarantine and social distancing till the end of the simulation time horizon. We did not vary the end date of the lockdown of Wuhan, since ending the lockdown policy prior to March 31, 2020 seemed unlikely given its central role in the early COVID-19 epidemic in China.

### Health outcomes and health care costs

Health consisted of cumulative number of SARS-CoV-2 infections and COVID-19 deaths in each of the four cities over the simulated time horizon. To be comparable with economic and mitigation costs, we monetized health outcomes using a disability-adjusted life-year (DALY) and willingness-to-pay approach. We estimated the DALYs associated with a fatal case of COVID-19 by calculated the years of life lost using the average life expectancy for each of the three age groups. For non-fatal SARS-CoV-2 infections, we calculated DALYs using estimates from the 2003 SARS outbreak in China [18] as disability weights for COVID-19 have not yet been established. To convert incurred DALYs into monetary units, we multiplied by a willingness-to-pay (WTP) threshold. In economic evaluations, it is typical to apply a WTP threshold per DALY averted of 1-3x national per-capita GDP [27]. Using the more generous 3x GDP for China in 2019, this corresponds to a WTP of $30,792 2020 USD per DALY averted [28].

In addition to health outcomes, we also considered direct health care costs associated with caring for hospitalized COVID-19 patients. The cost per hospitalized patient was estimated from the cost of a hospitalized SARS patient in the 2003 outbreak, inflated to 2020 USD ($4,125) [18].

Total disease burden for a given scenario was calculated as the sum of direct health care costs and monetized DALYs.

### Economic and mitigation costs

To estimate the economic cost of city-wide quarantine (Wuhan) and social distancing measures (other cities), we divided the loss in Q1 2020 GDP (accounting for both GDP reductions and foregone projected growth relative to Q1 2019) in each city by the duration of the policy (69 days in Wuhan; 38 days in other cities) to calculate the city-specific daily economic cost. For Wuhan, this resulted in a daily city-wide quarantine cost of 340 million 2020 USD; while social distancing incurred a daily cost of 232, 349, 377 million 2020 USD in Chongqing, Beijing, and Shanghai, respectively (Table 2). This reflects the heterogeneity in each city’s productivity and impact of social distancing (or city-wide quarantine for Wuhan) on its economy. Additional details about how economic losses were calculated can be found in the Additional file 1 — Technical Appendix.

**Table 2:** Estimated economic losses (in billion 2020 USD) for each modeled city

Sources	Wuhan	Chongqing	Beijing	Shanghai
Total economic loss	$23.46	$8.85	$13.27	$14.32
Total daily loss	$ 0.340	$0.232	$0.349	$0.377

The cost of travel-history based quarantine and contact tracing in cities other than Wuhan was based on the per-person cost of contact tracing for other communicable diseases $16.438 USD/day from Armruster *et al* [17]. This cost was applied to all individuals with recent travel from Wuhan.

We calculate the overall cost for a given city under a given mitigation scenario as the sum of the economic loss, mitigation costs, and monetized health burden. This analysis assumes a societal perspective. Costs were not discounted due to the short simulation time horizon (four months).

## Results

### Model calibration and validation

Through model calibration, our model was able to closely match the number of (diagnosed) infections in the “Elderly” age category and the total number of deaths reported in Wuhan on Mar 9, 2020, by the Chinese Center for Disease Control and Prevention [11], Table 3. The calibrated per-contact transmission rate was 3.90% (95% credible interval (95% CI): 3.86% to 3.94%). Our estimates were consistent with an early WHO report estimating that 1% to 5% contacts of infected individuals were also diagnosed with SARS-CoV-2 infection [29]. Informed by case information from the Diamond Princess Cruise [30], we also estimated the mean proportion of infections that are symptomatic to be 29.1% – 66.6%, depending on age category (Table 1). Similarly, using mortality data from a recent epidemiological study on COVID-19 outbreak in Wuhan, China [31] as prior information, we found through Bayesian calibration that mortality rates for each age category, shown in Table 1. We simulated the status quo scenario through the end of our time horizon, Mar 31, 2020, to validate the model against actual epidemiological data. In comparing the cumulative deaths predicted by the model in the four cities, we found that it was well-matched mortality rates in Wuhan as well as Shanghai and Beijing. Our predicted mortality in Chongqing was somewhat higher than the observed data, however, the number of deaths over this time period was small and subject to uncertainty (details in Additional file 1 — Technical Appendix, Section 4).

**Table 3:** Model outputs as compared to calibration target data

Target description	Value	Model output - mean (95% credible interval)	Source
**Calibration targets**			
Number of clinically confirmed COVID-19 cases aged 60 years or older in Wuhan on Mar 9, 2020	15,384	15,573 (15,337 - 15,819)	Chinese CDC [11]
Reported number of COVID-19 deaths in Wuhan as of Mar 9 2020	2,404	2,404 (2,274 - 2,530)	Chinese CDC [11]
Proportion of reported COVID-19 deaths as of Feb 11 2020, by age			Chinese CDC [12]
0-19 years old	0.1%	0.08% (0.01% - 0.18%)	
20-59 years old	18.9%	18.88% (18.05% - 19.52%)	
60+ years old	81.0%	81.04% (80.30% - 81.94%)	

### Varying mitigation strategy start date

**Fig. 3:**
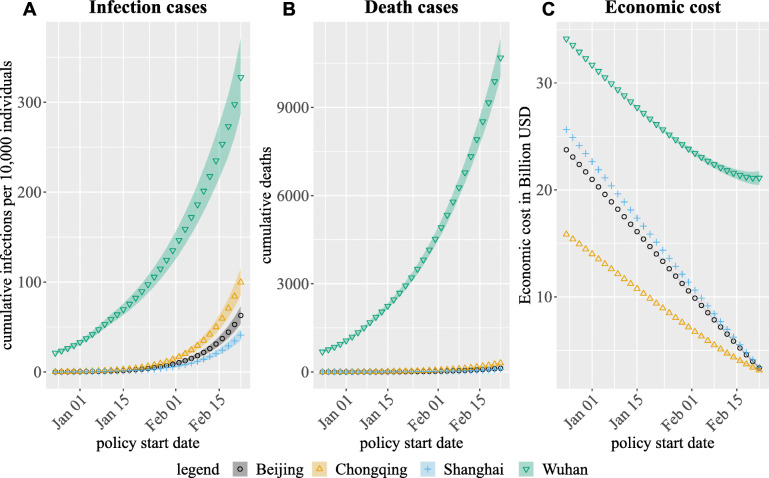
Mean (marker) and 95% credible intervals (shaded area) of model-predicted outcomes when varying the start for all control measures. In the status quo, all control measures were implemented on Jan 23, 2020. For all scenarios, we assumed workforce (“Adult”) social distancing in cities other than Wuhan ended on Feb 29, 2020. All other control measures were assumed to continue through Mar 31, 2020

Our model predicts that implementing all three mitigation strategies two weeks earlier (on Jan 9, 2020) would have averted 47% (1525) of COVID-19 deaths in Wuhan and over 70% of deaths in the other three cities (20 in Chongqing, 8 in Beijing, 6 in Shanghai) (Table 4 and Fig. 3). However, this would come at an increase in economic costs of 14% (3.71 billion USD) in Wuhan and over 35% in the other three cities (3.19, 4.85, 5.24 billion USD in Chongqing, Beijing, Shanghai respectively). Implementing mitigation strategies just one week earlier was predicted to avert 35% (664) of deaths in Wuhan (over 50% in the other three cities, 7 in Chongqing and 2 in Beijing, 2 in Shanghai) with an increase in economic costs of only 7% (1.79 billion USD) (16-18% in the other three cities, 1.58, 2.43, 2.63 billion USD in Chongqing, Beijing, Shanghai respectively). Delaying mitigation strategies by one week was predicted to decrease economic costs by the same amount (1.59, 1.55, 2.41, 2.61 in Wuhan, Chongqing, Beijing, Shanghai respectively), but would also result in 35% more deaths (1127) in Wuhan and more than 80% more deaths (23 in Chongqing, 9 in Beijing, and 7 in Shanghai) in the other three cities.

**Table 4:** Model-predicted outcomes when varying the start for all control measures. In the status quo, all control measures were implemented on Jan 23, 2020. For all scenarios, we assumed workforce (“Adults”) social distancing in cities other than Wuhan ended on Feb 29, 2020. All other control measures were assumed to continue through Mar 31, 2020. Outcomes are aggregated from Dec 1, 2019 through Mar 31, 2020

Control measure start date	Wuhan	Chongqing	Beijing	Shanghai
**Total COVID-19 deaths - mean (95% credible interval)**				
Jan 9 2020	1684 (1615 - 1750)	6 (6 - 6)	3 (2 - 3)	2 (2 - 2)
Jan 16 2020	2348 (2250 - 2442)	13 (12 - 13)	5 (5 - 6)	4 (4 - 4)
Jan 23 2020	3209 (3061 - 3348)	26 (25 - 27)	11 (10 - 11)	8 (8 - 8)
Jan 30 2020	4336 (4132 - 4530)	49 (47 - 52)	20 (19 - 22)	15 (14 - 16)
**Total cumulative SARS-CoV-2 infections per 10,000 - mean (95% credible interval)**				
Jan 9 2020	53.03 (48.71 - 57.60)	1.35 (1.21 - 1.50)	0.85 (0.76 -0.95)	0.56 (0.50 - 0.62)
Jan 16 2020	72.53 (66.12 - 79.33)	2.99 (2.66 - 3.34)	1.87 (1.66 - 2.10)	1.23 (1.09 - 1.37)
Jan 23 2020	97.25 (87.97 - 107.11)	6.31 (5.58 - 7.11)	3.92 (3.45 - 4.42)	2.56 (2.25 - 2.89)
Jan 30 2020	129.72 (116.46 - 143.88)	12.64 (11.08- 14.33)	7.81 (6.82 - 8.88)	5.10 (4.46 - 5.79)
**Total economic cost (billion USD) - mean (95% credible interval)**				
Jan 9 2020	$29.34 (29.28 - 29.40)	$12.13 (12.13 - 12.13)	$18.17 (18.17 - 18.17)	$19.60 (19.60 - 19.60)
Jan 16 2020	$27.44 (27.34 - 27.51)	$10.53 (10.53 - 10.54)	$15.75 (15.75 - 15.75)	$16.99 (16.99 - 16.99)
Jan 23 2020	$25.64 (25.50 - 25.76)	$8.94 (8.93 - 8.95)	$13.32 (13.32 - 13.32)	$14.36 (14.36 - 14.36)
Jan 30 2020	$24.04 (23.84 - 24.22)	$7.39 (7.37 - 7.41)	$10.91 (10.90 - 10.91)	$11.75 (11.74 - 11.75)

Overall, the model predicted that net economic cost (sum of economy losses and disease burden) would actually be lower in the four modeled cities if mitigation strategies had started later than the status quo. Economic costs would be minimized if mitigation strategies had been enacted on Feb 20, 2020 for Wuhan, and Feb 22, 2020 for three other cities respectively. In Wuhan, in particular, though substantial health benefits could be achieved through earlier mitigation (6,664 fewer deaths, 199.25 fewer infections per 10,000 individuals when starting on Jan 23 versus Feb 20, 2020), the economic loss of city-wide quarantining was so high that it outweighed these benefits (additional 4.54 billion USD when starting on Jan 23 versus Feb 20, 2020, Technical Appendix — Fig S1). However, if the economic cost of Wuhan’s quarantine was lower, equal to 1x or 2x what we estimate the cost of social distancing in Wuhan would be ($97.4 million USD per day) based on its share of national GDP, our model predicted an optimal mitigation start date of Jan 23, 2020 and Feb 10, 2020, respectively (Technical Appendix — Fig S1).

### Varying mitigation strategy end date

While the three cities we modeled (Chongqing, Beijing, and Shanghai) started to reopen workplaces on Feb 29, 2020, other cities and regions made different decisions. Some reopened workplaces, while schools remained closed. Others opted to extend school and workplace closures through mid-April [24,32]. We therefore explored how model-predicted epidemiological and economic outcomes would vary across five scenarios with different combinations of social distancing end dates for “Youth” and “Adult” age categories (Table 5). In all scenarios, we assumed that the “Elderly” age category would maintain social distancing practices (i.e., 50% reduction in daily contacts) through Mar 31, 2020 given their high risk of morbidity and mortality. In each of these scenarios, we only applied the social distancing end dates to cities other than Wuhan, and assumed that Wuhan would remain quarantined and fully engaged in social distancing. We made this assumptions because, as the epicenter of the epidemic, Wuhan remained under restrictions much longer than others cities and only gradually resumed economic and social activity starting on April 8, 2020 [26], well after our simulated end horizon.

**Table 5:** Scenarios with different social distancing end dates by age in cities other than Wuhan. In all scenarios, social distancing for all age categories was assumed to begin on Jan 23, 2020. Scenario 3 corresponds to the status quo

Social distancing end date scenarios			
	“Youth”	“Adult”	“Elderly”
Scenario 1	Feb 15	Feb 15	Mar 31
Scenario 2	Fed 29	Feb 29	Mar 31
Scenario 3	Mar 31	Feb 29	Mar 31
Scenario 4	Mar 31	Mar 31	Mar 31

Comparing Scenario 1 and 2, ending social distancing practice for non-elderly age groups two weeks earlier would have nearly tripled (doubled) the number of infections (deaths) in each of the three cities (Table 6). Further extending school closure for four weeks (Scenario 3, status quo, vs. Scenario 2) further reduced the number of infections (a reduction of 2.49, 1.69, 1.09 infections per 10,000 individuals in Chongqing, Beijing, Shanghai respectively), but had little impact on the number of predicted deaths (2, 1, 0 in Chongqing, Beijing, Shanghai respectively). Economic costs increased slightly (less than 0.01 billion USD, (0.1% of total economic costs) in all three cities) if only school closure was extended. Extending workforce closure for another four weeks (Scenario 4 vs. Scenario 3) resulted in similar reductions in infection and death cases as school closure, but doubled the economic cost (7.18, 10.91, 11.67 billion USD in Chongqing, Beijing, Shanghai respectively).

**Table 6:** Model-predicted outcomes under different end-date scenarios of school and workplace closures. Scenario 1: reopen schools and workplaces on Feb 15. Scenario 2: reopen schools and workplaces on Feb 15. Scenario 3 (status quo): reopen schools on Mar 31, workplaces on Feb 29. Scenario 4: reopen schools and workplaces on Mar 31. The “Elderly” are assumed to maintain social distancing through Mar 31. Outcomes are aggregated from Dec 1, 2019 through Mar 31, 2020

	Chongqing	Beijing	Shanghai
**Total COVID-19 deaths - mean (95% credible interval)**			
Scenario 1	45 (42 - 48)	19 (18 - 20)	14 (13 - 15)
Scenario 2	27 (25 - 28)	12 (11 - 12)	8 (8 - 9)
Scenario 3	26 (25 - 27)	11 (10 - 11)	8 (8 - 8)
Scenario 4	24 (23 - 26)	10 (10 - 11)	8 (7 - 8)
**Total cumulative SARS-CoV-2 infections per 10,000 individuals - mean (95% credible interval)**			
Scenario 1	24.71 (21.19 - 28.55)	14.95 (12.81 - 17.29)	9.60 (8.23 - 11.10)
Scenario 2	8.57 (7.49 - 9.73)	5.10 (4.46 - 5.80)	3.31 (2.90 - 3.77)
Scenario 3	6.31 (5.58 - 7.11)	3.92 (3.45 - 4.42)	2.56 (2.25 - 2.89)
Scenario 4	3.82 (3.45 - 4.21)	2.23 (2.01 - 2.46)	1.47 (1.33 - 1.62)
**Total economic cost, in billion USD - mean (95% credible interval)**			
Scenario 1	$5.83 (5.80 - 5.87)	$8.48 (8.47 - 8.50)	$9.12 (9.11 - 9.13)
Scenario 2	$8.94 (8.94 - 8.95)	$13.31 (13.31 - 13.32)	$14.36 (14.35 - 14.36)
Scenario 3	$8.94 (8.93 - 8.95)	$13.32 (13.32 - 13.32)	$14.36 (14.36 - 14.36)
Scenario 4	$16.12 (16.12 - 16.13)	$24.13 (24.13 - 24.13)	$26.03 (26.03 - 26.03)

**Fig. 4:**
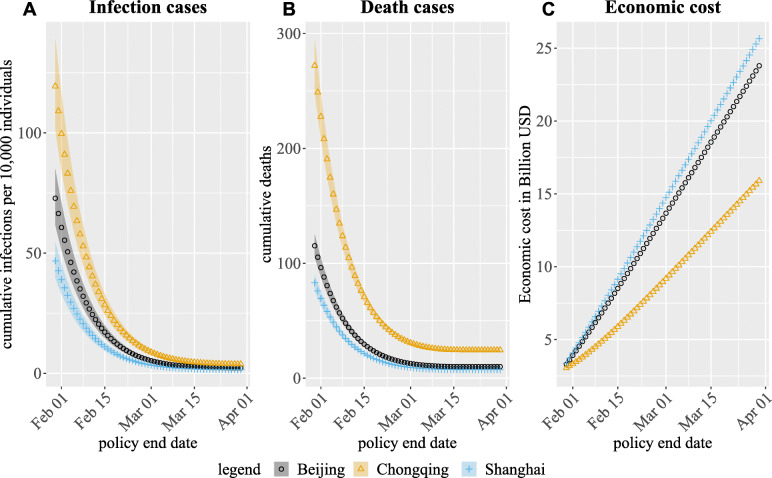
Mean (marker) and 95% credible intervals (shaded area) of model-predicted outcomes when enacting a uniform end date of social distancing measures for all age groups

We also compared results in Table 6 with uniform social distancing end dates, shown in Fig. 4. Ending social distancing practices for everyone on Feb 18 resulted in a similar number of deaths, as in Scenario 1 (social distancing for the “Youth” and “Adult” age groups ends on Feb 15). Ending social distancing for everyone on Mar 9 resulted in a similar number of deaths as in Scenario 3 (status quo). Similar findings apply to any one of the first three scenarios. These comparisons indicate that the value of social distancing is primarily driven by ubiquitous social distancing practice, rather than age-targeted control measures. However, the economic costs of the uniform social distancing end date are higher, as workplace closures result in major productivity losses.

### Sensitivity analysis

In the sensitivity analysis, we first explored the differential impact of implementing the different mitigation strategies at different times. We then conducted one-way sensitivity analyses on assumed model parameters, including the effectiveness of travel history screening, contact rate increases during Chinese New Year, and contact rate reduction when practicing social distancing (Additional file 1 — Technical Appendix, Section 1).

Among all mitigation strategies, we found that social distancing had the highest impact on the number of infections and deaths. The cumulative number of infections and deaths grow near-exponentially in all four cities with delayed social distancing (Technical Appendix — Fig S2 and Technical Appendix — Fig S3). However, when the start dates of Wuhan travel history screening and/or the Wuhan city-wide quarantine are delayed, infections and deaths in the other three cities increase, but not nearly as quickly (Technical Appendix — Fig S5 and Technical Appendix — Fig S6). This is because restricting and monitoring travels from Wuhan slows the importation of new cases to other cities, but does nothing to slow the local spread of undetected infections within other cities. Lastly, we found that economic losses were predominantly due to the daily costs of social distancing or city-wide quarantine rather than disease burden. This is consistent with our findings when varying strategy start dates.

One- and two-way sensitivity analyses varying key model parameters are presented in the Appendix, Section 3. When we vary the contact rate during Chinese New Year (CNY)/*chunyun* (from Jan 10 to Feb 18, 2020) in Technical Appendix — Fig S8, we find that both the epidemiological and economic outcomes increase exponentially with the CNY contact rate in Wuhan, while in other cities this (increasing) relationship is quasi-linear. This is because in the status quo, Wuhan city-quarantine and travel history screening efficiently prevented the growth of epidemic in other cities. As a consequence, varying contact rates during CNY resulted in a much larger impact in Wuhan, but not other cities. For the same reason, if contact rate reduction (when practicing social distancing) varied from 25% to 75%, we observed an exponential decrease in number of infections, deaths and economic cost in Wuhan (Technical Appendix — Fig S9). The changes were less steep in other places compared to Wuhan. Lastly, we observe from Technical Appendix — Fig S10 that model outcomes were not sensitive to the effectiveness of travel history screening in identifying all recent travelers from Wuhan.

Two-way sensitivity analyses (Technical Appendix — Fig S11 to Technical Appendix — Fig S16) confirmed our observations above. A higher contact rate during CNY, and/or a higher contact rate reduction when practicing social distancing could defer the optimal social distancing end date to a later date. Travel-history screening effectiveness was not a significant factor in determining the optimal policy end dates.

## Discussion

In this study, we provided insights on differential impact on timing and duration of mitigation strategies to address the COVID-19 outbreak in Wuhan (the epicenter of the outbreak), and three other highly-connected megacities: Chongqing, Beijing, and Shanghai. Our model predicted that instituting mitigation strategies earlier and extending them for longer reduces infections and deaths, but at a substantial economic cost. Of the three mitigation strategies considered, social distancing was the most effective, while the city-wide quarantine on Wuhan and travel history screening had only marginal impacts. When we considered age-targeted social distancing strategies, workplace closures to reduce contact among the “Adult” age group averted the greatest number of deaths, but also incurred the highest economic costs. Extending social distancing among “Youth” and “Elderly” age groups resulted in relatively few reductions in deaths.

By considering both health and economic impacts, our results highlight the trade-offs in deciding both when and for how long to institute mitigation strategies. Our model predicted that enacting mitigation strategies later than the status quo would have reduced economic costs. For Wuhan, this finding is largely driven by the extremely high cost of the city-wide quarantine, which had only a modest impact on preventing infections, especially when compared to social distancing. Thus, it is clear that China made large economic sacrifices to achieve control over the epidemic, perhaps even greater sacrifices than would be justified by the number of averted COVID-19 infections and deaths under typical economic evaluation standards [33]. However, decision-making in emergency contexts will inevitably involve some inefficiencies given the scant information available at the time the decision must be made. Furthermore, our economic projections should be interpreted cautiously, as they only account for within-city outcomes and do not include the financial ramifications to other cities and countries arising from imported infections. As Wuhan is a transportation hub for the country with the potential to disperse infections throughout the country, a city-specific perspective under-estimates the secondary benefits. Additionally, we may have under-estimated the economic value of an averted SARS-CoV-2 infection, even if non-fatal, as there is growing evidence of debilitating long-term sequelae that persists even after recovery [34*–*36].

Our study has several limitations. First, our study was limited in scope, focusing on Wuhan as the epicenter epidemic and travelers out of Wuhan into three major cities. We did not consider inter-city travel beyond these cities, nor the possibility of infections being introduced from a city other than Wuhan. This assumption is most valid early on in the epidemic [7]. Given the small numbers of infections that occur in the three cities outside of Wuhan, it is unlikely that the re-introduction of cases from these cities would have substantially altered Wuhan’s epidemic trajectory, particularly given travel restrictions under a city-wide quarantine. Had we substantially deviated from the status quo mitigation strategies and timing, a more comprehensive model of Chinese travel patterns may have been necessary. Second, we chose to project outcomes over a relatively limited time horizon (until the end of March). Projecting epidemic outcomes too far into the future can be challenging, as policy decisions may change and be difficult to predict. Furthermore, new interventions, treatments, and prevention measures, may become available that would substantially alter long-term outcomes of the epidemic and in turn, the optimal policy. Third, we only stratified the population based on age as we primarily focused on age-specific mitigation policies; other factors like gender and presence of co-morbidities may influence the severity and mortality of COVID-19 cases [37,38]; to the extent cities differ in these features, we did not capture these differences. As data become available, our modeling framework could be extended to account for further population heterogeneity. Fourth, our compartmental modeling approach simplifies contact patterns within the population. A more complex simulation modeling approach, such as agent-based modeling, could be parameterized to reflect more realistic mixing structures, such as stratifying and clustering interactions occurring in households, at schools, and at work places. However, this added realism comes at the expense of greater computational burden as well as requiring more detailed data to parameterize these mixing behaviors. Lastly, we only approximated the impact of strained health care resources on COVID-19 mortality by assuming a higher mortality rate in Wuhan than in the other three cities. If we extended our analysis scenarios resulting in more widespread infection in cities outside of Wuhan, it would be important to model cityspecific healthcare capacity and downstream impacts on COVID-19 outcomes more explicitly.

## Supplementary Information

**Additional file 1** **Technical appendix — fig s2.** Varying social distancing start date only, maintaining initiation of other control measures on Jan 23, 2020.
**Technical appendix — fig s3.** Varying social distancing start date only, maintaining initiation of other control measures on Jan 23, 2020. Wuhan is excluded from the plot to show greater detail in the other three cities.
**Technical appendix — fig s4.** Varying start date for all control measures and doubled mortality rate for all age groups, maintaining initiation of other control measures on Jan 23, 2020.
**Technical appendix — fig s5.** Varying Wuhan travel history screening start date, maintaining initiation of other control measures on Jan 23, 2020. Wuhan is excluded from the plot to show greater detail in the other three cities.
**Technical appendix — fig s6.** Varying Wuhan quarantine and Wuhan travel history screening start date, maintaining social distancing initiation on Jan 23, 2020. Wuhan is excluded from the plot to show greater detail in the other three cities.
**Technical appendix — fig s7.** Varying Wuhan travel history screening and social distancing start date, maintaining Wuhan city-wide quarantine initiation on Jan 23, 2020. Wuhan is excluded from the plot to show greater detail in the other three cities.
**Technical appendix — fig s8.** One-way sensitivity analysis on contact rate during Chinese New Year (CNY). In the base case, we assumed contacts rates increase by 20% for all age categories.
**Technical appendix — fig s9.** One-way sensitivity analysis on the relative contact rate reduction when practicing social distancing. In the base case, this is assumed to be 0.50.
**Technical appendix — fig s10.** One-way sensitivity analysis on travel history screening effectiveness, *α*. In the base case, *α*=1.0.
**Technical appendix — fig s11.** Two-way sensitivity analysis (subplot 1) on contact rate during Chinese New Year (CNY) and travel history screening effectiveness, *α*. For this subplot, *α*=1.0.
**Technical appendix — fig s12.** Two-way sensitivity analysis (subplot 1) on contact rate during Chinese New Year (CNY) and travel history screening effectiveness, *α*. For this subplot, *α*=0.75.
**Technical appendix — fig s13.** Two-way sensitivity analysis (subplot 1) on contact rate during Chinese New Year (CNY) and travel history screening effectiveness, *α*. For this subplot, *α*=0.50.
**Technical appendix — fig s14.** Two-way sensitivity analysis (subplot 1) on contact rate during Chinese New Year (CNY) and relative contact rate reduction when practicing social distancing. For this subplot, contact rate reduction is 25%.
**Technical appendix — fig s15.** Two-way sensitivity analysis (subplot 1) on contact rate during Chinese New Year (CNY) and relative contact rate reduction when practicing social distancing. For this subplot, contact rate reduction is 50%.
**Technical appendix — fig s16.** Two-way sensitivity analysis (subplot 1) on contact rate during Chinese New Year (CNY) and relative contact rate reduction when practicing social distancing. For this subplot, contact rate reduction is 75%.

## Data Availability

The simulation source code can be found on Github at: https://github.com/Anthony-zh-Zhang/COVID_19_model.
