# Supplementary material for: Optimal timing and effectiveness of COVID-19 outbreak responses in China: a modelling study
Source: BMC Public Health. 2022 Apr 7;22:679. doi: 10.1186/s12889-022-12659-2 (PMC8989110; doi:10.1186/s12889-022-12659-2)
Supplement: Supplementary file 1 — Additional file 1 Technical appendix — fig s2. Varying social distancing start date only, maintaining initiation of other control measures on Jan 23, 2020. Technical appendix — fig s3. Varying social distancing start date only, maintaining initiation of other control measures on Jan 23, 2020. Wuhan is excluded from the plot to show greater detail in the other three cities. Technical appendix — fig s4. Varying start date for all control measures and doubled mortality rate for all age groups, maintaining initiation of other control measures on Jan 23, 2020. Technical appendix — fig s5. Varying Wuhan travel history screening start date, maintaining initiation of other control measures on Jan 23, 2020. Wuhan is excluded from the plot to show greater detail in the other three cities. Technical appendix — fig s6. Varying Wuhan quarantine and Wuhan travel history screening start date, maintaining social distancing initiation on Jan 23, 2020. Wuhan is excluded from the plot to show greater detail in the other three cities. Technical appendix — fig s7. Varying Wuhan travel history screening and social distancing start date, maintaining Wuhan city-wide quarantine initiation on Jan 23, 2020. Wuhan is excluded from the plot to show greater detail in the other three cities. Technical appendix — fig s8. One-way sensitivity analysis on contact rate during Chinese New Year (CNY). In the base case, we assumed contacts rates increase by 20% for all age categories. Technical appendix — fig s9. One-way sensitivity analysis on the relative contact rate reduction when practicing social distancing. In the base case, this is assumed to be 0.50. Technical appendix — fig s10. One-way sensitivity analysis on travel history screening effectiveness, α. In the base case, α=1.0. Technical appendix — fig s11. Two-way sensitivity analysis (subplot 1) on contact rate during Chinese New Year (CNY) and travel history screening effectiveness, α. For this subplot, α=1.0. Technical appendix — fig s12. Tw [file 12889_2022_12659_MOESM1_ESM.pdf]

S1 Text: Technical Appendix
Optimal timing and effectiveness of COVID-19 outbreak responses in China: a modelling study

Anthony Zhenhuan Zhang, Eva A. Enns

1 Model framework

1.1 Wuhan model

We developed the following Susceptible-Exposed-Infected-Recovered (S, E, I, R respectively) dynamic compartmental model to simulate COVID-19 outbreak in Wuhan, China since December 1, 2019:

$$\begin{aligned}
dS_i(t) =& - S_i(t)\Big(\beta_I \sum_j C_{ij}(t)\frac{I_j^S(t)+I_j^A(t)}{N_j(t)} + \frac{z(t)}{\sum_j N_j(t)}\Big) \\
&+ \frac{T^{(c,w)}(t)}{\sum_j N_j(t)}N_i(t) - \frac{T^{(w,c)}(t)}{\sum_j N_j(t)}S_i(t) \\
dE_i(t) =& + S_i(t)\Big(\beta_I \sum_j C_{ij}(t)\frac{I_j^S(t)+I_j^A(t)}{N_j(t)} + \frac{z(t)}{\sum_j N_j(t)}\Big) \\
&- \frac{E_i(t)}{D_E} - \frac{T^{(w,c)}(t)}{\sum_j N_j(t)}E_i(t) \\
dI_i^S(t) =& + \frac{E_i(t)}{D_E}\rho_i - \frac{I_i^S(t)}{D_I} - \mu_i I_i^S(t) - \frac{T^{(w,c)}(t)}{\sum_j N_j(t)}I_i^S(t) \\
dI_i^A(t) =& + \frac{E_i(t)}{D_E}(1-\rho_i) - \frac{I_i^A(t)}{D_I} - \frac{T^{(w,c)}(t)}{\sum_j N_j(t)}I_i^A(t) \\
dR_i(t) =& + \frac{I_i^A(t)+I_i^S(t)}{D_I} - \frac{T^{(w,c)}(t)}{\sum_j N_j(t)}R_i(t) \qquad (1)
\end{aligned}$$

In the equations above, i represents one of the following age categories: 0-19 years old ("Youth"), 20-59 years old ("Adults"), and 60 years old and older ("Elderly"). $N_i(t)$ is the population size for age category i in Wuhan on day t. Population size ($N_i(t)$) is time-dependent because of the exchange of population through daily inbound and outbound travels. There are two possible sources of infection: either through person-to-person contacts with individuals in the asymptomatic infection state I^A, or the symptomatic infection state I^S with rate β_I. $C_{ij}(t)$ is the averaged daily contact counts that individuals in age category i made with individuals in age category j on day t. ρ_i is the proportion of infections that are symptomatic in age category i. We describe the estimation procedure for contact matrix in 1.3. For the Wuhan model only, a non-zero zoonotic force of infection was included for the time that the seafood market thought to be the origin of the outbreak was still operational (Dec 1, 2019 through Jan 1, 2020). As in Wu et al. [1], we assumed this force of infection to be 86 cases per day prior to the market's closure. The zoonotic force of infection is represented by $z(t)$,

which is set to 86 per day until the Huanan seafood market closure on Jan 1, 2020, at which point it drops to zero. In the model, death could only occur for those in the symptomatic infection state, I^S. The death rate was denoted by μ_i for age category i, reflecting substantial age-dependent differences in COVID-19 mortality. $T^{(c,w)}(t)$ ($T^{(w,c)}(t)$) represents the inbound (outbound) travel to (from) Wuhan on day t. Notation is summarized in S2 Table and a model state transition diagram is shown in Fig 1.

The model was used to track daily numbers of infections and deaths, as well as the distribution of outbound travelers leaving Wuhan across the disease and age compartments. The number of travelers arriving at each city from Wuhan and arriving in Wuhan from other cities was calculated from mobility data from Baidu, a Chinese internet search engine company [2]. Our approach is conceptually consistent with using O-D matrix for mobility modeling in other COVID-19 modelling literature [3].

In both Wuhan model and Other-city model, we assumed in the base case, transmission rate β_I is the same for symptomatic and asymptomatic cases. While it is the case for many illnesses that a symptomatic person is more infectious than an asymptomatic person, viral load studies of people infected with SARS-CoV-2 have found that symptomatic and asymptomatic people have similar levels of viral shedding [18]. In addition, it is now understood that SARS-CoV-2 spreads primarily via aerosols, which are emitted in everyday actions like breathing and talking [19, 20]. While symptomatic cases are more likely to emit large droplets through coughing and sneezing, these expiratory activities are not essential for SARS-CoV-2 transmission. Furthermore, a symptomatic person is more likely to stay home because they feel sick, whereas an asymptomatic person will continue their regular activities in the absence of frequent, routine testing.

1.2 Other-city model

We also modeled outcomes in three other Chinese cities (Beijing, Shanghai, and Chongqing), accounting for the introduction of COVID-19 cases from Wuhan during the early phase of the epidemic. The disease progression model for these three Chinese cities followed the same basic structure as the Wuhan model, except that additional stratifications were included to track individuals with recent travel history from Wuhan and recent travelers under quarantine (when applicable). Each compartment was therefore subscripted by both age group, i, and population, k, where $k = W$ for those recently arriving from Wuhan and $k = L$ for the local population without recent Wuhan travel. To incorporate the effects of travel history screening policies (and quarantine) in these other cities, we have a parameter $\alpha(t)$ that reflects the effectiveness with which travel history screening identifies travelers arriving from Wuhan. In the base case, we assume 100% effectiveness ($\alpha = 1$). To simulate a scenario where travel history screening is enacted at time t_{THS}, we divert α proportion of arrivals from Wuhan to quarantined states from $t = t_{THS}$ onward. Disease dynamics are represented in Fig 2 and follow the following system of differential equations:

$$
\begin{aligned}
dS_{ik}(t) =& - S_{ik}(t)\Bigg(\beta_I \sum_j C_{ij} \frac{\sum_k I^S_{jk}(t) + I^A_{jk}(t)}{\sum_k N_{jk}(t)}\Bigg) \\
&+ \mathbf{1}\{k = w\}(1 - \alpha(t))S_i^{(w,c)}(t)p_{wl} \\
dE_{ik}(t) =& + S_{ik}(t)\Bigg(\beta_I \sum_j C_{ij} \frac{\sum_k I^S_{jk}(t) + I^A_{jk}(t)}{\sum_k N_{jk}(t)}\Bigg) \\
&- \frac{E_{ik}(t)}{D_E} + \mathbf{1}\{k = w\}(1 - \alpha(t))E_i^{(w,c)}(t)p_{wl} \\
dI^S_{ik}(t) =& + \frac{E_{ik}(t)}{D_E}\rho_i - \frac{I^S_{ik}(t)}{D_I} - \mu_i I^S_{ik}(t) + \mathbf{1}\{k = w\}(1 - \alpha(t))I_i^{S,(w,c)}(t)p_{wl} \\
dI^A_{ik}(t) =& + \frac{E_{ik}(t)}{D_E}(1-\rho_i) - \frac{I^A_{ik}(t)}{D_I} + \mathbf{1}\{k = w\}(1 - \alpha(t))I_i^{A,(w,c)}(t)p_{wl} \\
dR_{ik}(t) =& + \frac{I^A_{ik}(t) + I^S_{ik}(t)}{D_I} + \mathbf{1}\{k = w\}(1 - \alpha(t))R_i^{(w,c)}(t)p_{wl}
\end{aligned} \tag{2}
$$

Once travel history screening is enacted, a set of quarantined compartments are added to the model to account for individuals with Wuhan travel history who are being isolated. We let $S_{i,q,t'}(t)$, $E_{i,q,t'}(t)$, $I^A_{i,q,t'}(t)$, $I^S_{i,q,t'}(t)$, $R_{i,q,t'}(t)$ represent individuals in compartments S, E, I^A, I^S, R and age category i who have been isolated for t' days on day t, respectively. Isolated individuals in the E', I^A and I^S compartments progress through the disease stages, ultimately recovering or dying, based on the following dynamics:

$$
\begin{aligned}
dE_{i,q,t'}(t) =& - \frac{E_{i,q,t'}(t)}{D_E} \\
dI^A_{i,q,t'}(t) =& + \frac{E_{i,q,t'}(t)}{D_E}(1-\rho_i) - \frac{I^A_{i,q,t'}(t)}{D_I} \\
dI^S_{i,q,t'}(t) =& + \frac{E_{i,q,t'}(t)}{D_E}\rho_i - \frac{I^S_{i,q,t'}(t)}{D_I} - \mu_i I^S_{i,q,t'}(t) \\
dR_{i,q,t'}(t) =& + \frac{I^A_{i,q,t'}(t) + I^S_{i,q,t'}(t)}{D_I}
\end{aligned} \tag{3}
$$

Individuals who enter quarantine in the susceptible compartment remain susceptible during their quarantine period, as we assume they have no contact with others. After the 14-day observation period (i.e., when $t' = 14$), all quarantined individuals except those in I^S compartment transition to the non-quarantined Wuhan-originating population. Patients in the I^S compartment remain quarantined until their illness is resolved, either through recovery or death.

1.3 Contact Matrix Estimation

We assumed that age-specific mixing patterns in China follow a preferential mixing structure. The number of contacts between age group i and age group j is then represented by the following equations:

$$
C_{ij}(t) = \Bigg(\gamma_i \mathbf{1}_{i=j} + (1-\gamma_i)\frac{(1-\gamma_j)M_j(t)N_j(t)}{\sum_k (1-\gamma_k)M_k(t)N_k(t)}\Bigg) M_i(t),
$$

where γ_i is the contact ratio reserved for peers in the same age category i, and $1 - \gamma_i$ is split among all groups. $M_i(t)$ is the daily number of contacts for individuals in age category i on day t. We estimated γ_i and M_i based on numbers and ages of contacts reported in a study of age-specific contact patterns in Southern China [4].

However, these mixing patterns might not be representative for Chinese New Year (CNY). During CNY, total number of daily contact might increase due to the large inter-generational social gatherings in celebrations of CNY from Jan 10 to Feb 18, 2020. We varied the rate of contacts increase during CNY in the sensitivity analysis (Section 3).

2 Cost estimation

2.1 Disease Burden

We measured the overall health impacts of COVID-19 using the disability-adjusted life-year (DALY) framework [5]. DALYs reflect both the years of life lost (YLL) due to premature mortality associated with a given health condition as well as the years lost due to disability (YLD) resulting from the condition. DALYs can be converted into a monetary disease burden by multiplying an appropriate willingness-to-pay (WTP), typically taken to be 1x - 3x Gross Domestic Product (GDP) per capita in the study region [6]. For our analysis in China, we used a WTP of 3x GDP per-capita in China in 2019 ($10,264): $30,792 USD [7].

The YLL due to a COVID-19 death was calculated by taking the difference between life expectancy in China and the average age of the population within each age category. Given a current life expectancy of 74.6 years [8] and the age composition of the Chinese population [9], we calculated the YLL to be 67.20, 37.11, and 9.45 for the "Youth", "Adult", and "Elderly" age categories, respectively. The cost per fatal case of COVID-19 was then calculated as WTP $\times$ YLL.

Because COVID-19 is so new, a condition-specific disability weight for the calculation of YLD is not available. We, therefore, followed the same methodology as an analysis of the DALYs associated with the 2003 SARS outbreak in China [10]. There, YLD were calculated by weighting the average number of workdays lost by age-specific productivity weights (0.15 for "Youth", 0.8 for "Adults", and 0.1 for the "Elderly"). For a non-fatal case of COVID-19, we assumed the number of workdays lost (from symptom onset through hospitalization and recovery) was similar as that reported SARS measures in Guangzhou, China, by Du et al. [10]: 31.28, 58.55, and 54.84 days for the "Youth", "Adult", and "Elderly" age categories, respectively. The cost per non-fatal case of COVID-19 was then calculated as

$$\text{cost per non-fatal case} = \text{WTP/day} \times \text{workdays lost} \times \text{productivity weight}$$

This monetary disease burden estimate was only applied to clinically confirmed cases (moderate to severe), as undetected mild cases were assumed to result in a negligible number of YLD.

The healthcare costs for supportive treatment of a clinically confirmed (moderate to severe) case of COVID-19 were assumed to be similar to that of SARS in China [10]. These healthcare costs were estimated to be $2,925 (in 2003 USD) per hospitalized case, which we inflated to $4,125 in 2020 USD using the Consumer Price Index.

2.2 Economy loss

Total economic loss under status quo mitigation strategies China reported a contraction of 6.8% in its first quarter GDP in 2020 due to COVID-19 [11]. Given the

Q1 GDP for China in 2019 [7,9] and the predicted GDP growth assuming no COVID-19 outbreak for 2020 [11], we estimated an economic loss (due to COVID-19) of $395.36 billion USD for Q1 in China. In the first quarter of 2019, Wuhan had a GDP of $48.68 billion USD, with a predicted growth rate of 7.5% to 7.8% in 2020 [12]. In the absence of the COVID-19 outbreak, the Q1 GDP of Wuhan would have been projected at $52.43 billion USD. With the COVID-19 outbreak, Wuhan experienced a 40.5% drop in the first quarter GDP in 2020 [21]. This results in an economic loss of $23.46 billion USD. We assume all other provinces share proportionally in the remaining percent loss of national GDP growth in Q1 2020. Based on each province's share of national GDP [7], we calculated the estimated GDP loss for Chongqing, Beijing, and Shanghai as $8.85, $13.27, $14.32 billion USD respectively.

Daily costs Wuhan's earliest return-to-work date was on April, 8, 2020 [13]. In other cities, industry return-to-work starts on Mar 1, while schools remain closed till Mid-March or Mid-April [14,15]. Although few industries have come back online since Feb 15, they only reached 30% to 50% of the usual production level [16]. We assume that the cost came of mass social distancing is primarily due to workplace closures. Then, we approximate the duration of mitigation strategy implementation as 69 days in Wuhan, and 38 days in other cities. We therefore estimated the daily economic losses to be $ 0.340, $0.232, $0.349, $0.377 billion USD in Wuhan, Chongqing, Beijing and Shanghai, respectively.

As a point of comparison, we calculated the expected social distancing cost for Wuhan in the absence of a city-wide quarantine (and if they had not been the focal point of the initial SARS-CoV-2 pandemic). This calculation followed the same procedure as that for Chongqing, Beijing and Shanghai. Prior to the COVID-19 outbreak, the GDP in Wuhan was 42% that of Chongqing's, with similar growth trajectories. We, therefore, estimated the daily loss due to social distancing (only) in Wuhan as $0.42 \times \$0.232$ billion USD = $0.0974 billion USD. We use this social distancing cost as a benchmark for considering different quarantine costs.

Sources	Wuhan	Chongqing	Beijing	Shanghai
Total economic loss	$23.46	$8.85	$13.27	$14.32
Total daily loss	$ 0.340	$0.232	$0.349	$0.377
Daily cost due to social distancing	$ 0.0974	$0.232	$0.349	$0.377

Table S1. Estimated economic losses (in billion USD) for each modeled city.

3 Sensitivity analysis results

3.1 Varying mitigation strategy timing

In this section, we examined the differential impact of implementing the different mitigation strategies at different times (i.e., additional counterfactual scenarios). End dates for all mitigation strategies were as in the status quo (Manuscript, Method Section). In S2 Fig, we varied the start date of mass social distancing in all cities. Cumulative infections and deaths were increasing exponentially with delayed social distancing. Economic costs in cities excluding Wuhan (S3 Fig) were decreasing with a delayed social distancing start date. This is because strategy implementation costs were the main component of the total economic cost, whereas the disease burden is relatively low in these three places. On the other hand, the economic cost in Wuhan is always increasing because of the relatively high disease burden. To account for the possibility

of under reporting, in In S4 Fig, we doubled the mortality rate and then varied social distancing start date. The conclusion was consistent with our observations and findings in S2 Fig, except that the date of minimum economic cost would have become sooner due to increased disease burden.

Varying the travel history screening start date along (S5 Fig) resulted in a near-linear increase in both epidemiological and economic outcomes before Jan 23, 2020, the travel history screening start date. From Jan 23 onward, the frequency of travels from Wuhan was low due to Wuhan quarantine. As a consequence, the increase in infection and death cases were minimal. When the start date of Wuhan quarantine was varied simultaneously (S6 Fig), the number of infections, deaths, and total economic costs were increasing with delayed policy start dates.

We did not vary the start date of Wuhan quarantine alone since Wuhan quarantine is not likely to be implemented solely in reality.

Compare all strategy scenarios involving social distancing practice (Fig 4, S3 Fig, and S7 Fig), we found that social distancing highest impact on numbers of infections and deaths, economic costs (due to disease burden) among all mitigation strategies.

3.2 Varying model parameters

In this section, we varied model parameters including travel history screening effectiveness α, contact rates during Chinese New Year, contact rates reduction during social distancing practice, as well as the per contact transmission rate β_E. We vary the contact rates during Chinese New Year to reflect the traditionally large inter-generational social gatherings for Chinese New Year celebrations. We use the status quo scenario as the base case in this Section.

When we vary the contact rate during Chinese New Year (CNY)/*chunyun* (from Jan 10 to Feb 18, 2020) in S8, we find that both the epidemiological and economic outcomes increase exponentially with the CNY contact rate in Wuhan, while in other cities this (increasing) relationship is quasi-linear. This is because in the status quo, Wuhan city-quarantine and travel history screening efficiently prevented the growth of epidemic in other cities. As a consequence, varying contact rates during CNY resulted in a much larger impact in Wuhan, but not other cities. For the same reason, if contact rate reduction (when practicing social distancing) varied from 25% to 75%, we observed an exponential decrease in number of infections, deaths and economic cost in Wuhan (S9). The changes were less steep in other places compared to Wuhan. Lastly, we observe from S10 that model outcomes were not sensitive to the effectiveness of travel history screening in identifying all recent travelers from Wuhan.

When varying contact rate during CNY and travel history screening effectiveness together (S11, S12, S13), for any given THS, contact rate increase during CNY would delay the optimal social distancing end date. This is simply because social distancing becomes more effective in this scenario. we did not observe significant changes while varying THS for any given contact rate increase in CNY.

When varying contact rate during CNY and relative contact rate reduction when practicing social distancing, we observed that both contact rate increase during CNY or contact rate reduction when practicing social distancing is highly influential on the optimal social distancing end dates. A higher contact rate during CNY, and/or a higher contact rate reduction when practicing social distancing could delay the optimal SD end date to a later date.

3.3 doubling mortality rates

We performed sensitivity analyses while varying start date of all control measures, with doubled mortality rates for all age groups. Results are shown in S4 Fig. Implementing

all three mitigation strategies two weeks earlier (on Jan 9, 2020) would have averted 47% of COVID-19 deaths in Wuhan and over 70% of deaths in the other three cities Implementing mitigation strategies just one week earlier was predicted to avert 26% of deaths in Wuhan (over 45% in the other three cities). Delaying mitigation strategies by one week resulted in 35% more deaths in Wuhan and more than 88% more deaths in the other three cities. On the other hand, the difference in economic cost becomes smaller. This is because increased disease burden mitigated the dominating cost of control measures. The date of minimum economic cost would have become sooner due to increased disease burden.

4 Status quo predictions

In Wuhan (under the status quo), our model predicted a total of 97.25 (95% CI: 87.97 - 107.11) cumulative infections per 10,000 individuals by Mar 31, 2020. Our model also predicted a total of 3,209 COVID-19 deaths (95% CI: 3,061 - 3,348) over the same time period, with 79.5% (95% CI: 77.9% - 81.2%) of these occurring in the "Elderly" age category. This is very close to total number of death: 3099, reported by [17] as of Mar 31, 2020.

We also predicted a total of 6.31 (95% CI: 5.58 - 7.11), 3.92 (95% CI: 3.45 - 4.42), 2.56 (95% CI: 2.25 - 2.89) cumulative infections per 10,000 individuals by Mar 31, 2020 in Chongqing, Beijing, and Shanghai, respectively. The model predicts 26 (95% CI: 25 - 27), 11 (95% CI: 10 - 11), 8 (95% CI: 8 - 8) deaths in Chongqing, Beijing, and Shanghai, respectively, by Mar 31, 2020. This is far fewer deaths than seen in Wuhan due to both lower disease burden and reduced mortality risk associated with infection in these cities. The actual death cases did not fall into the confidence interval but still very close to the predicted number: 9 death cases (7 deaths cases) in Beijing (Shanghai) respectively. This may because of the small sample size. The economic cost under status quo mitigation measures, including both economic losses and disease burden costs, was estimated to be $25.64 (95% CI: 25.50 - 25.76) billion USD in Wuhan from Dec 1, 2019 through Mar 31, 2020, primarily due to prolonged social distancing. In other cities, we predicted net economic cost of $8.94 (95% CI: 8.93 - 8.95), $13.32 (95% CI: 13.32 - 13.32), $14.36 (95% CI: 14.36 - 14.36) billion USD in Chongqing, Beijing, and Shanghai, respectively. Economic losses were predominantly due to the daily costs social distancing or city-wide quarantine (for Wuhan) rather than disease burden.

5 Tables and figures

Model Notation Summary	
	general notations
N_i	(alive) population size in age category i
D_I	infectious period
D_E	incubation period
β_I	per contact transmission rate in state I^S or I^A
C_{ij}	average daily contact that an individual in age category i made with individuals in age agroup j
μ_i	mortality rate for age category i
	notation for Wuhan Model
S_i	susceptible individuals in age category i
E_i	exposed individuals in age category i
I_i^A	infected and asymptomatic individuals in age category i
I_i^S	infected and symptomatic individuals in age category i
R_i	recovered individuals in age category i
D_i	dead individuals in age category i
z	zoonotic force of infection, same as in Wu et al. [1]
$T^{c,w}$	daily inbound travel
$T^{w,c}$	daily outbound travel
	notation for Other-city Model
S_{ik}	susceptible individuals in age category i and population k
E_{ik}	exposed individuals in age category i and population k
I_{ik}^A	infected and asymptomatic individuals in age category i and population k
I_{ik}^S	infected and symptomatic individuals in age category i and population k
R_{ik}	recovered individuals in age category i and population k
D_{ik}	dead individuals in age category i and population k
$S_i^{(w,c)}$	imported Wuhan susceptible individuals in age category i
$E_i^{(w,c)}$	imported Wuhan exposed individuals in age category i
$I_i^{S,(w,c)}$	imported Wuhan infected and symptomatic individuals in age category i
$I_i^{A,(w,c)}$	imported Wuhan infected and asymptomatic individuals in age category i
$R_i^{(w,c)}$	imported Wuhan recovered individuals in age category i
p_{wl}	proportion of exportation from Wuhan to the local city

Table S2. Model Notation Summary

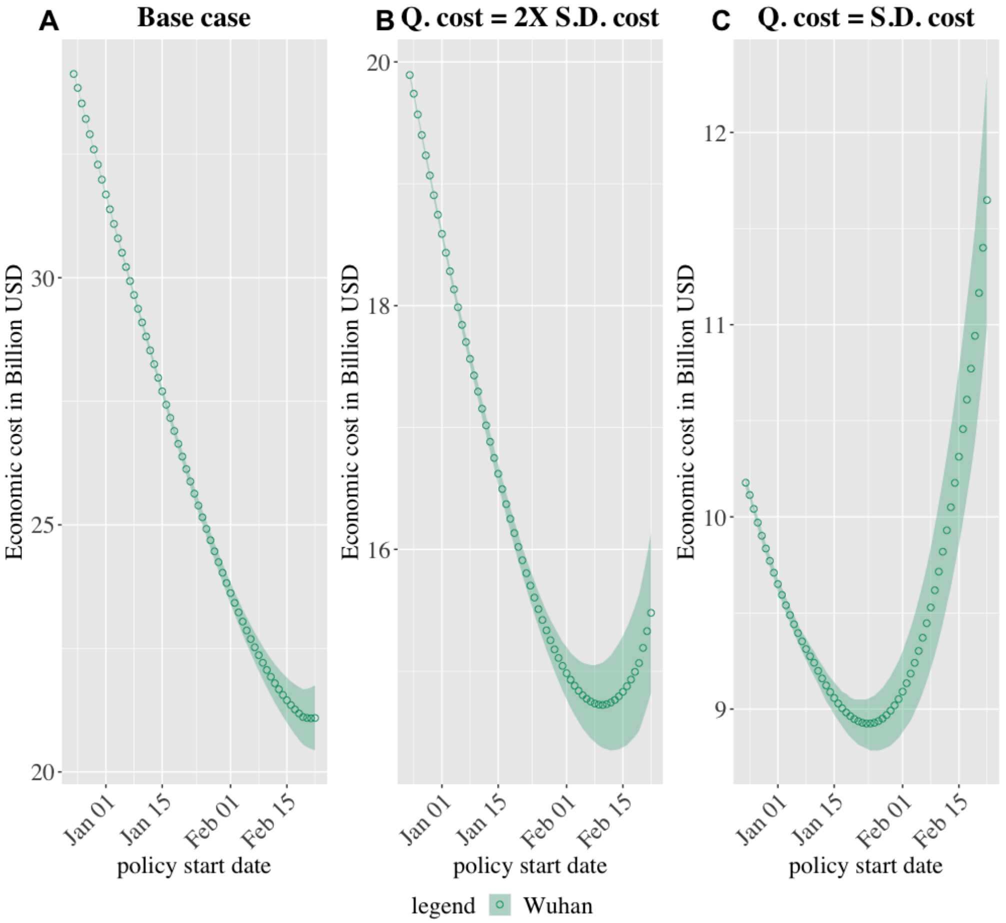

Fig S1. One-way sensitivity analysis on Wuhan quarantine cost while varying all mitigation strategy start date simultaneously. Panel A corresponds to the base case. Q. cost = daily quarantine cost, S.D. cost = daily social distancing cost

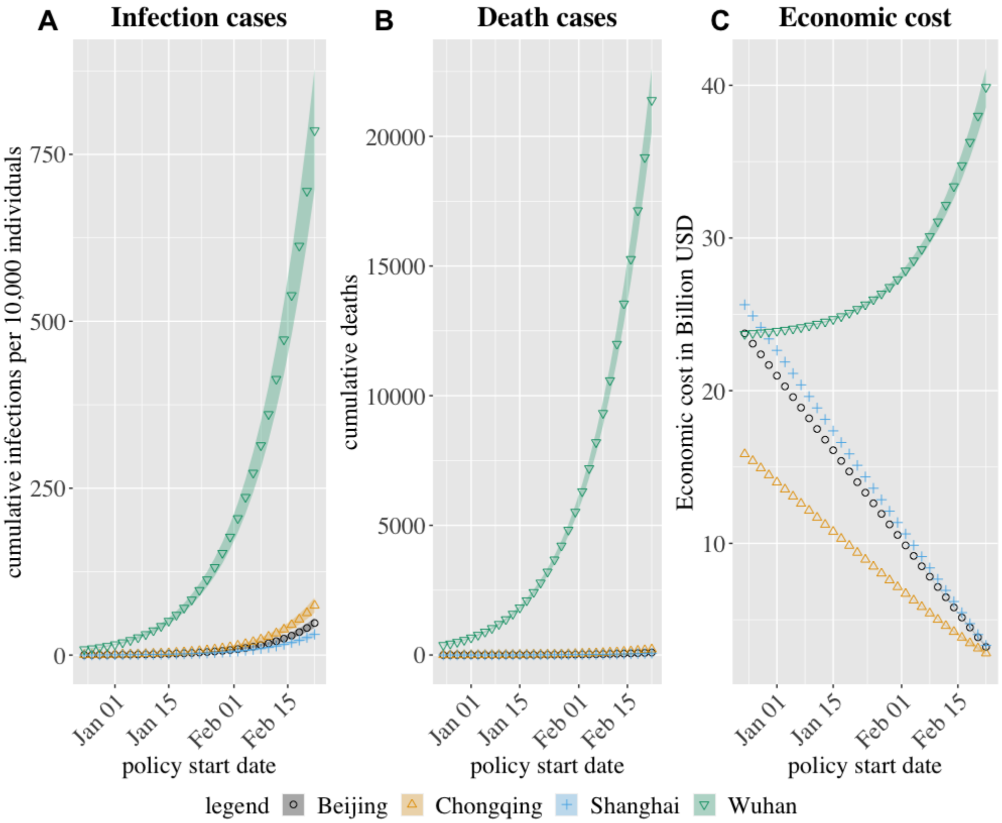

Fig S2. Varying social distancing start date only, maintaining initiation of other control measures on Jan 23, 2020.

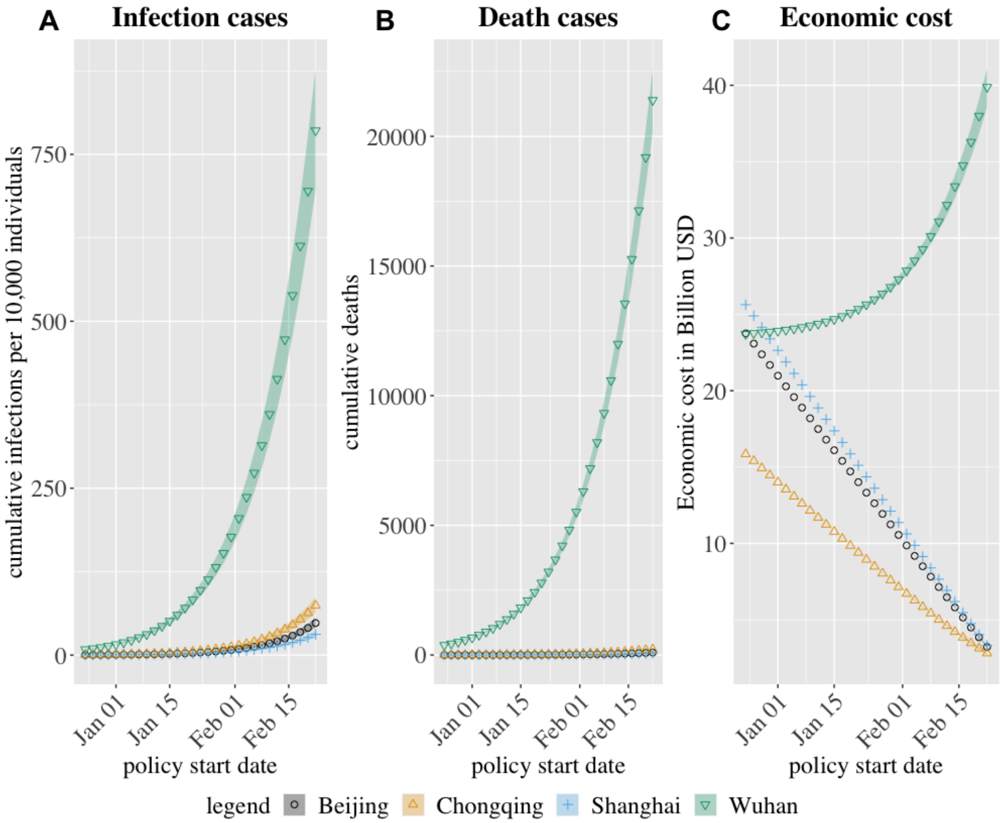

Fig S3. Varying social distancing start date only, maintaining initiation of other control measures on Jan 23, 2020. Wuhan is excluded from the plot to show greater detail in the other three cities.

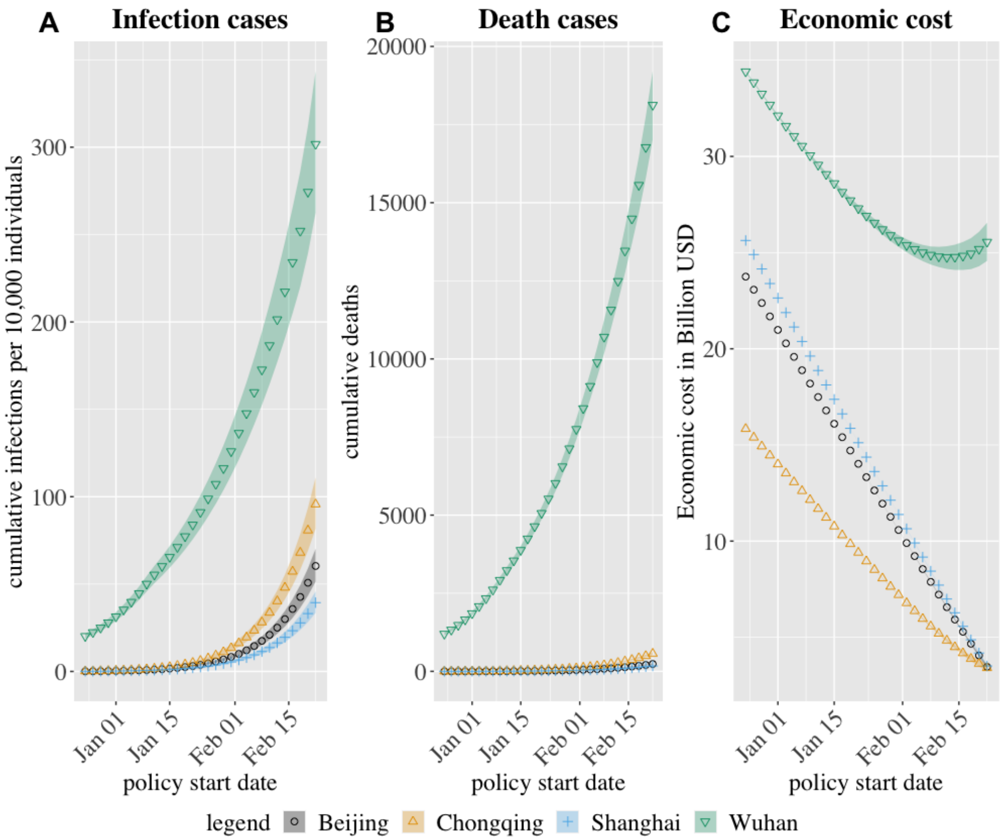

Fig S4. Varying start date for all control measures and doubled mortality rate for all age groups, maintaining initiation of other control measures on Jan 23, 2020.

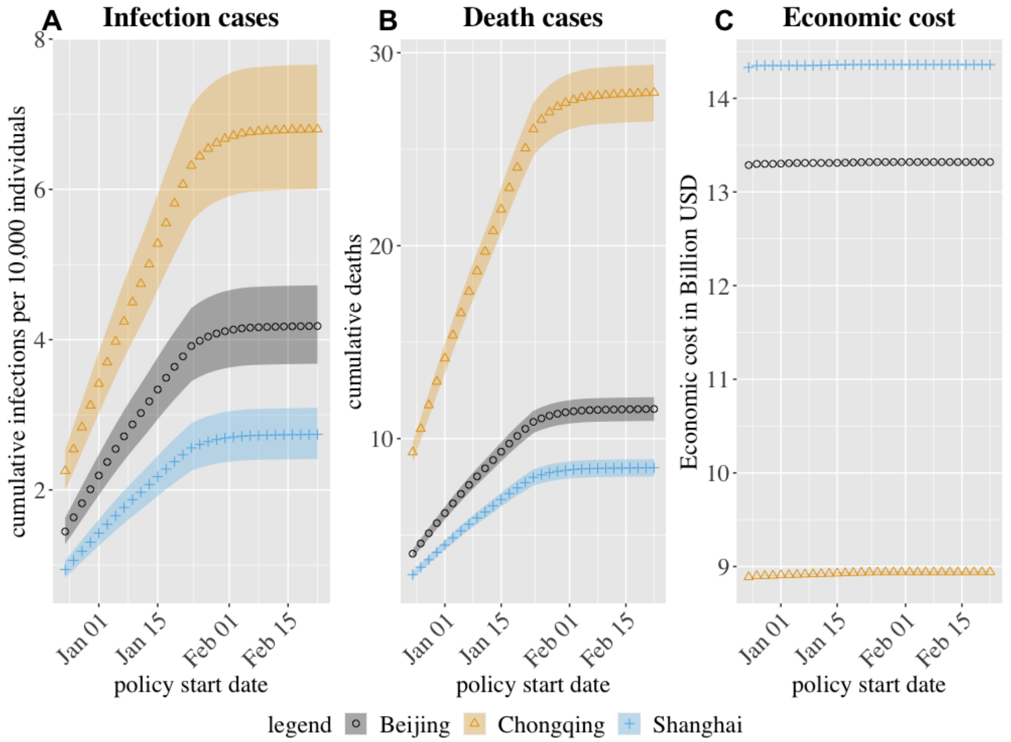

Fig S5. Varying Wuhan travel history screening start date, maintaining initiation of other control measures on Jan 23, 2020. Wuhan is excluded from the plot to show greater detail in the other three cities.

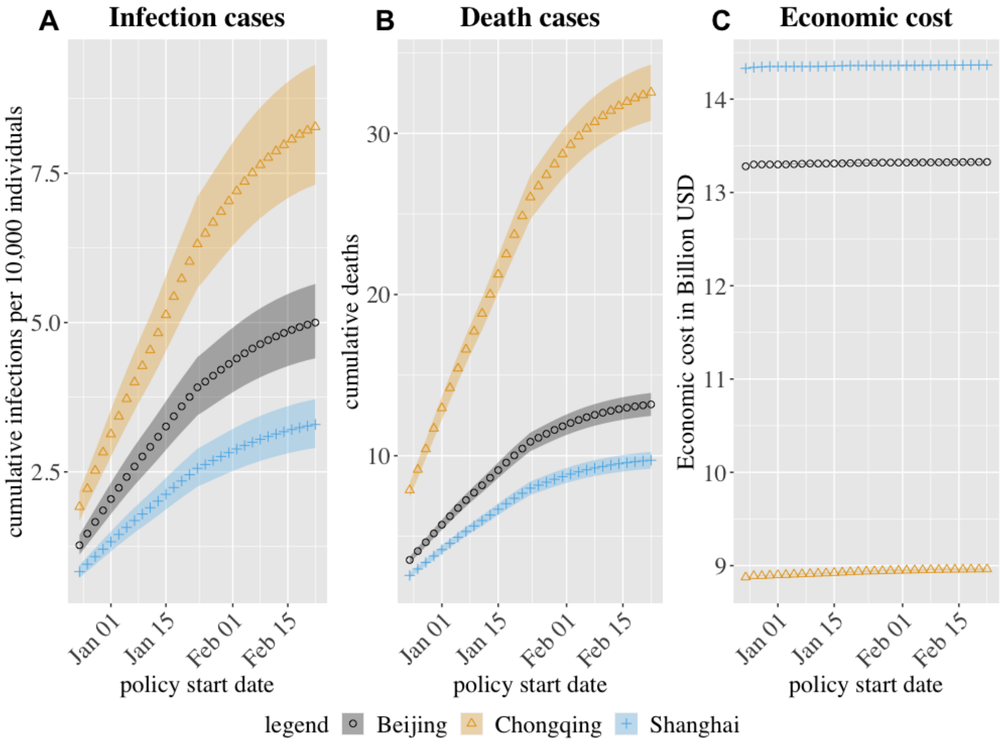

Fig S6. Varying Wuhan quarantine and Wuhan travel history screening start date, maintaining social distancing initiation on Jan 23, 2020. Wuhan is excluded from the plot to show greater detail in the other three cities.

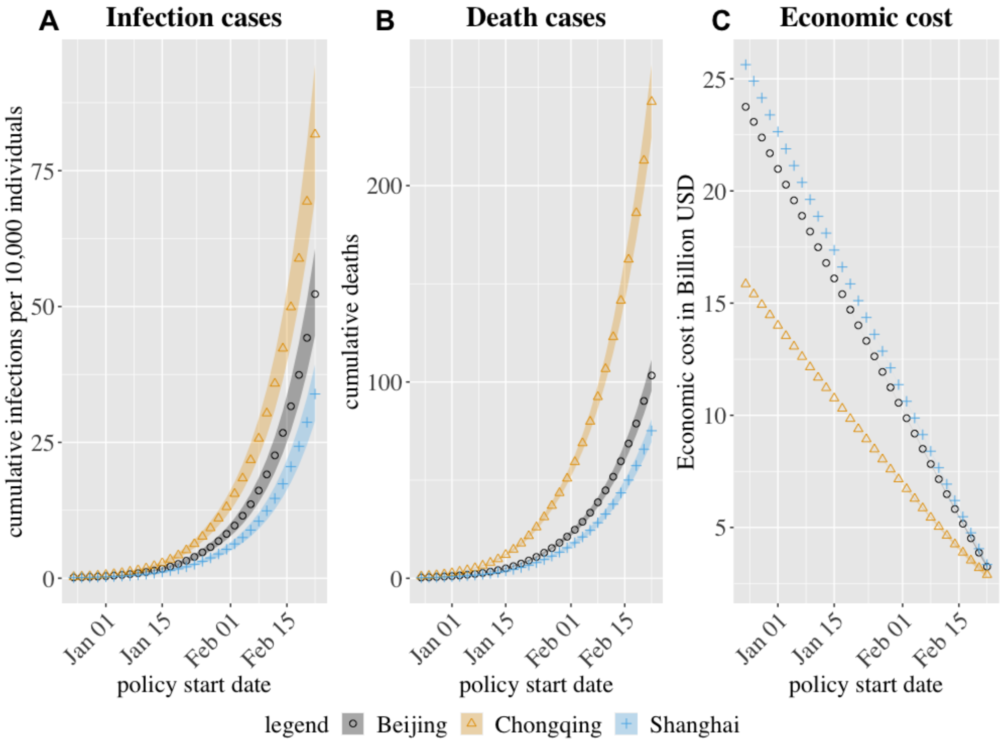

Fig S7. Varying Wuhan travel history screening and social distancing start date, maintaining Wuhan city-wide quarantine initiation on Jan 23, 2020. Wuhan is excluded from the plot to show greater detail in the other three cities.

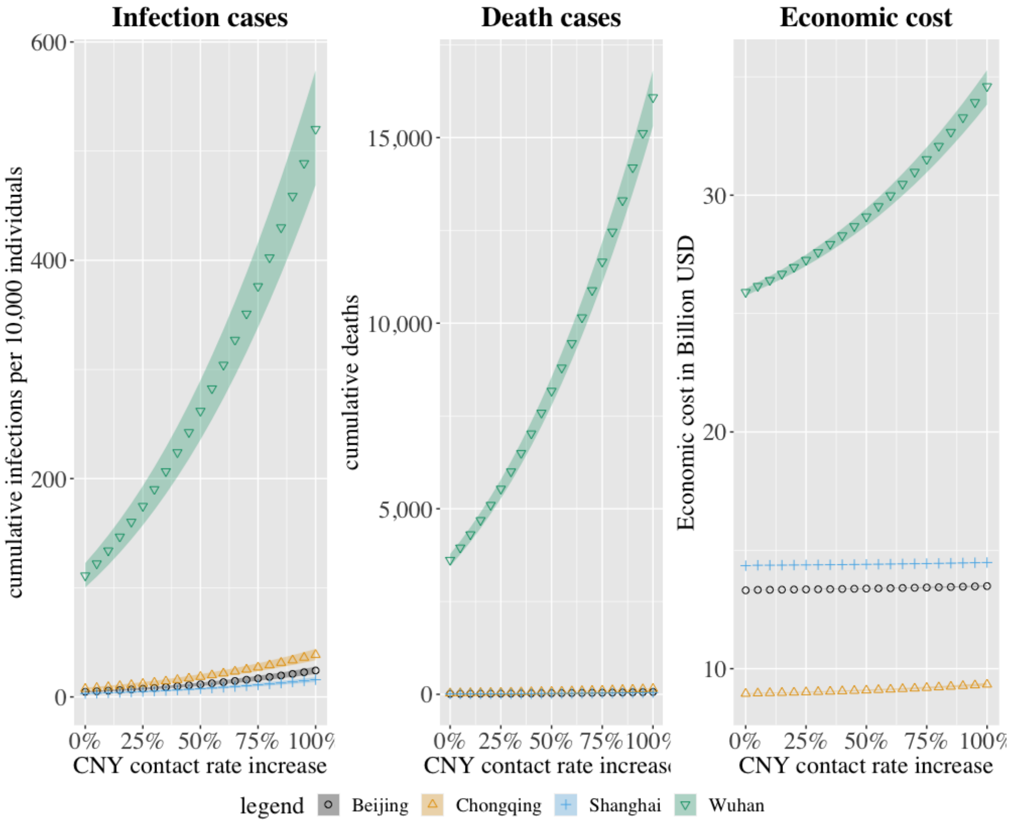

Fig S8. One-way sensitivity analysis on contact rate during Chinese New Year (CNY). In the base case, we assumed contacts rates increase by 20% for all age categories.

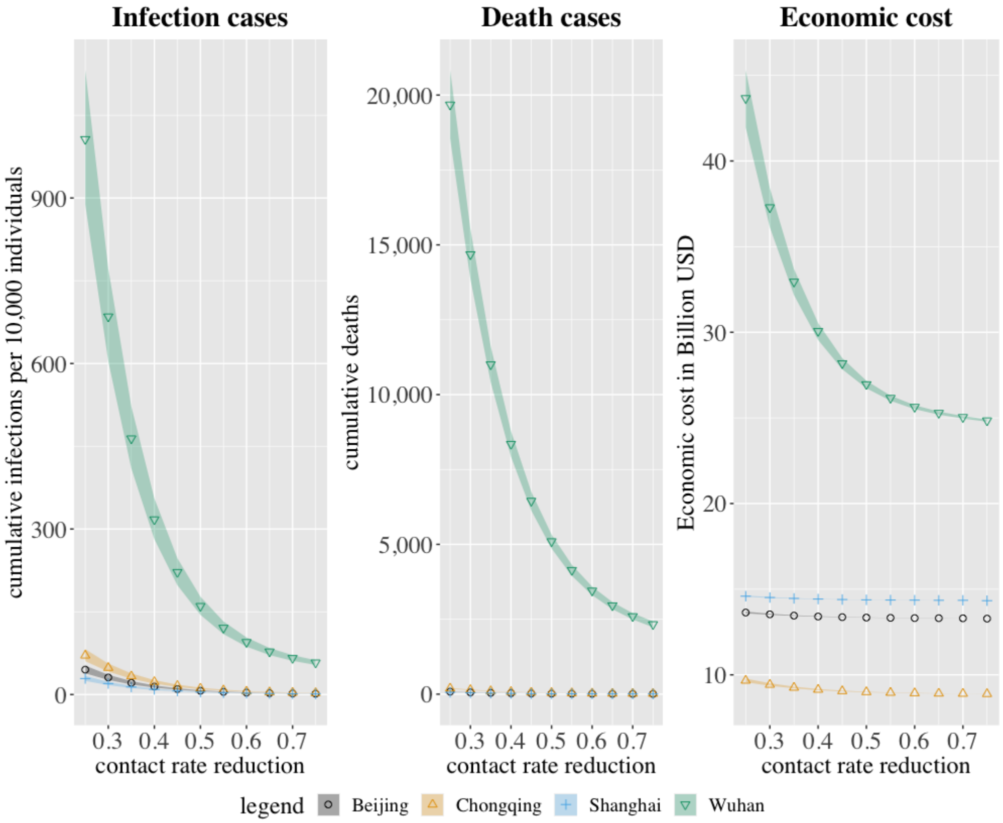

Fig S9. One-way sensitivity analysis on the relative contact rate reduction when practicing social distancing. In the base case, this is assumed to be 0.50.

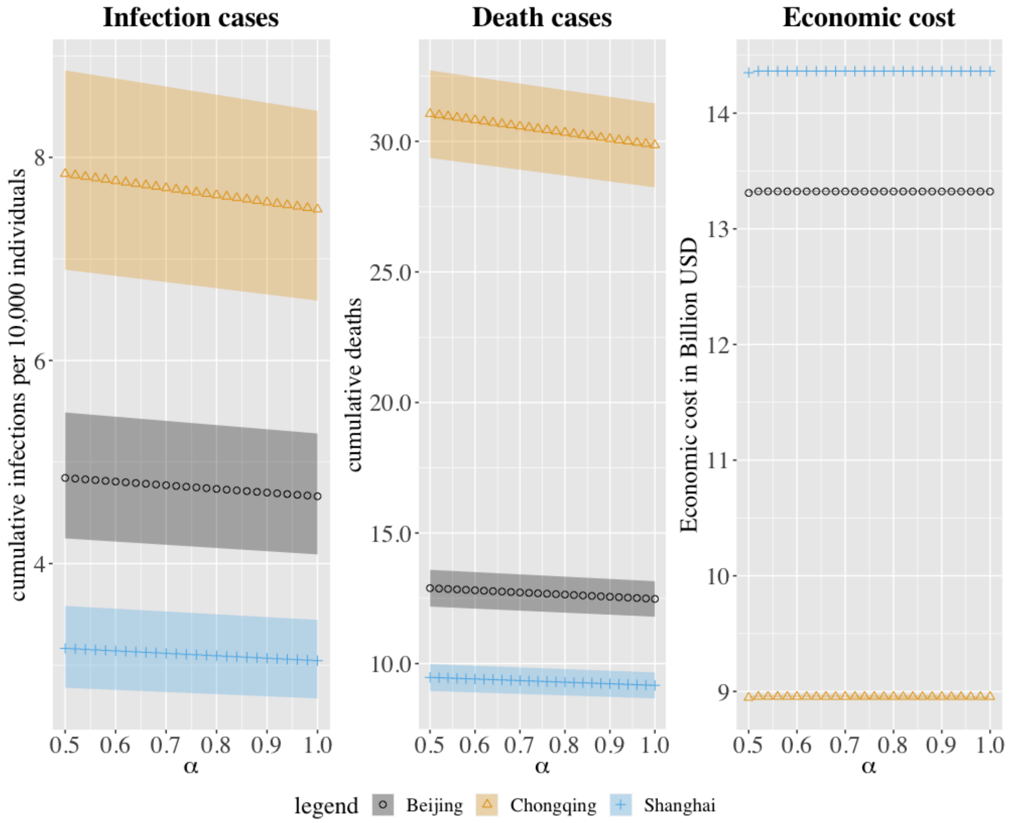

Fig S10. One-way sensitivity analysis on travel history screening effectiveness, α. In the base case, $\alpha = 1.0$.

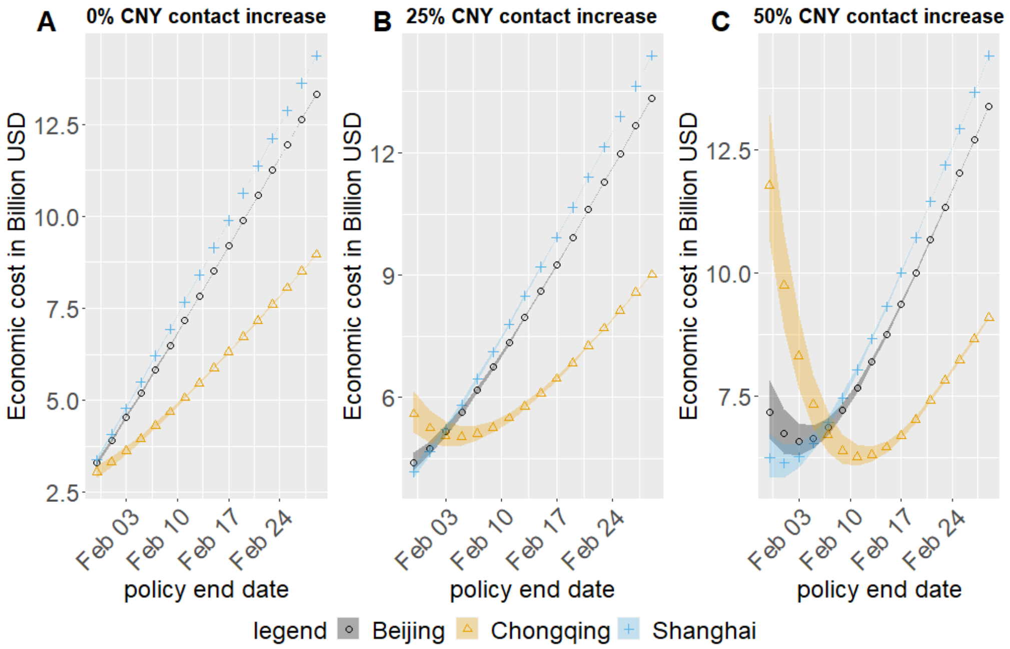

Fig S11. two-way sensitivity analysis (subplot 1) on contact rate during Chinese New Year (CNY) and travel history screening effectiveness, α. For this subplot, $\alpha = 1.0$.

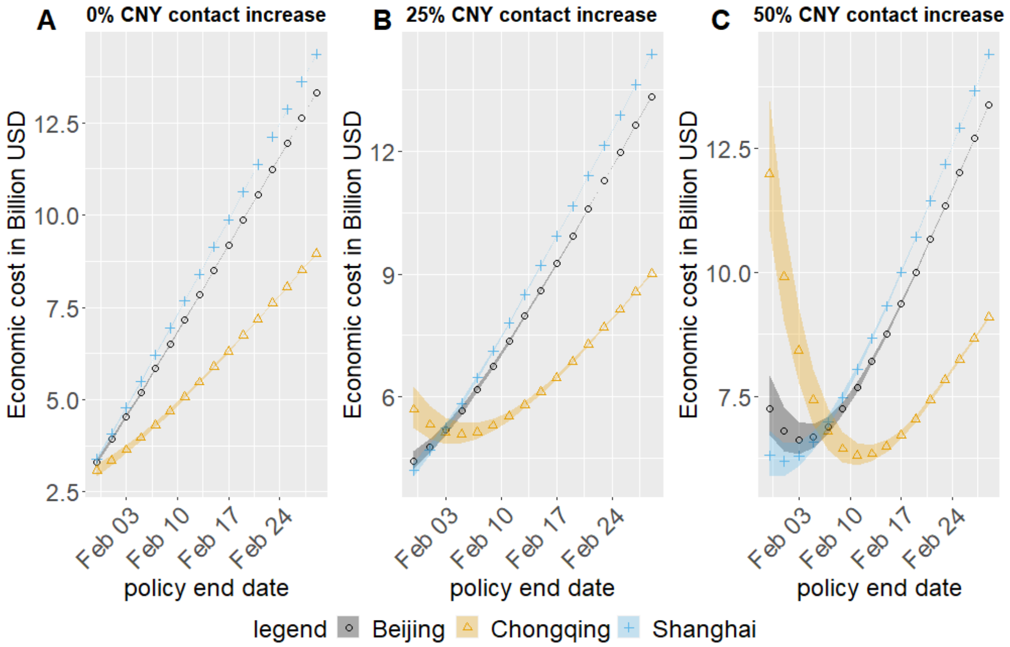

Fig S12. two-way sensitivity analysis (subplot 1) on contact rate during Chinese New Year (CNY) and travel history screening effectiveness, α. For this subplot, $\alpha = 0.75$.

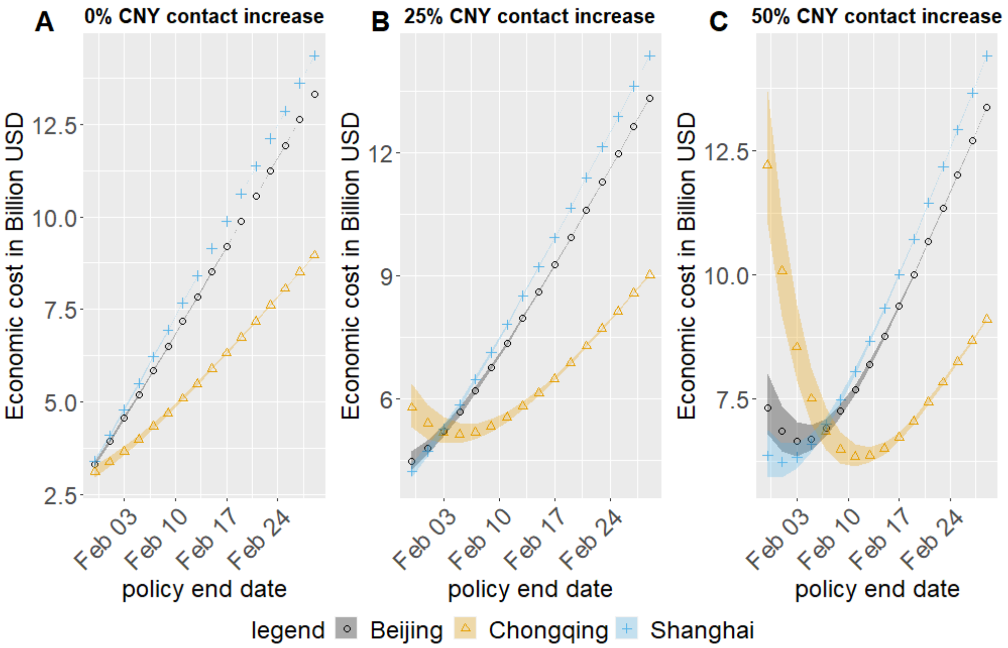

Fig S13. two-way sensitivity analysis (subplot 1) on contact rate during Chinese New Year (CNY) and travel history screening effectiveness, α. For this subplot, $\alpha = 0.50$.

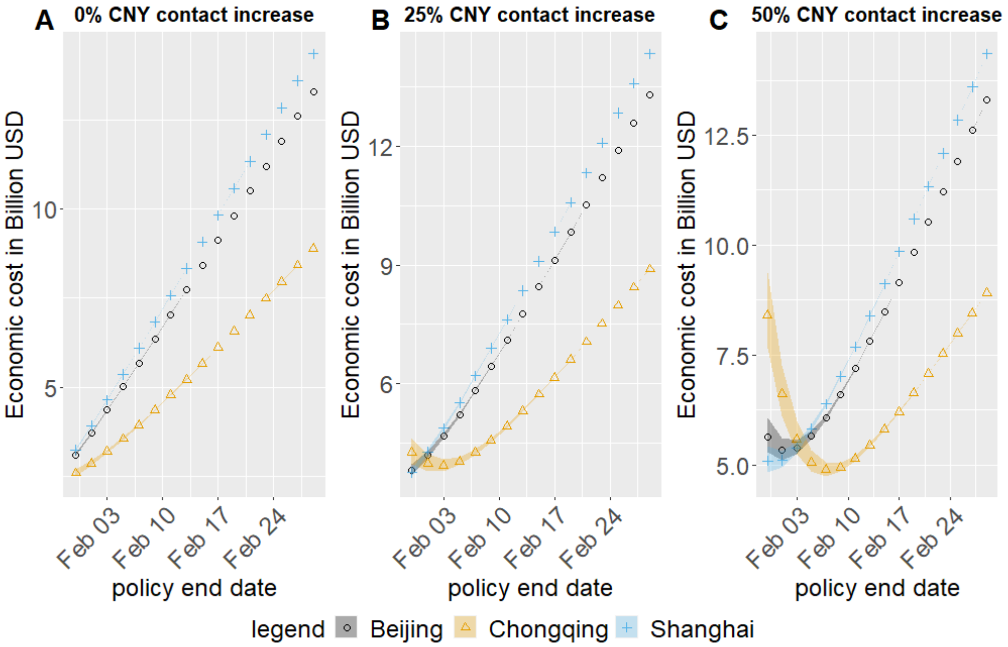

Fig S14. two-way sensitivity analysis (subplot 1) on contact rate during Chinese New Year (CNY) and relative contact rate reduction when practicing social distancing. For this subplot, contact rate reduction is 25%.

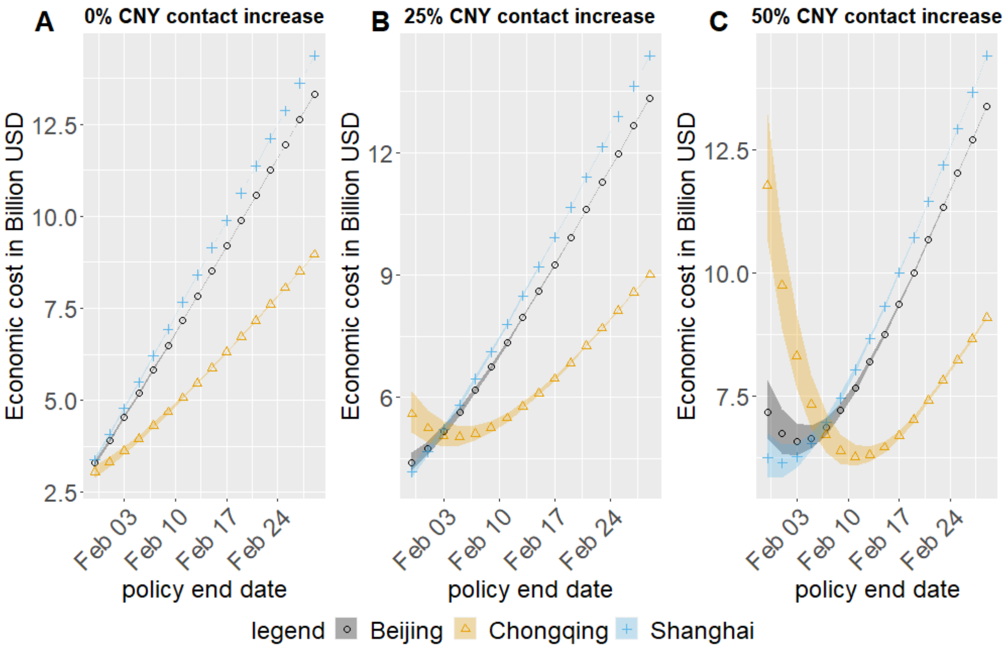

Fig S15. two-way sensitivity analysis (subplot 1) on contact rate during Chinese New Year (CNY) and relative contact rate reduction when practicing social distancing. For this subplot, contact rate reduction is 50%.

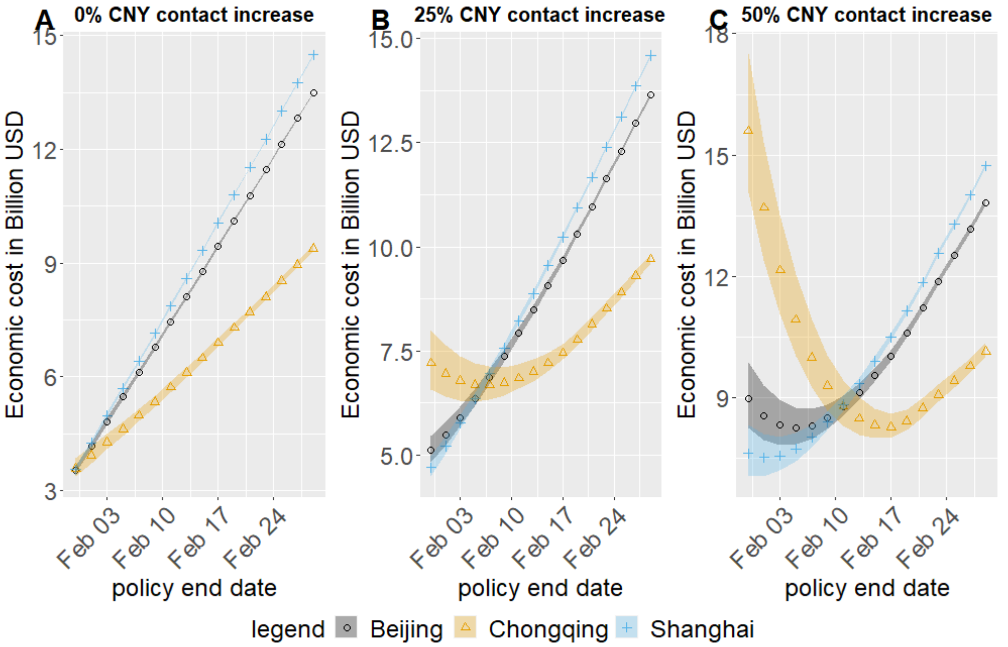

Fig S16. two-way sensitivity analysis (subplot 1) on contact rate during Chinese New Year (CNY) and relative contact rate reduction when practicing social distancing. For this subplot, contact rate reduction is 75%.

References

1. Wu JT, Leung K, Leung GM. Nowcasting and forecasting the potential domestic and international spread of the 2019-nCoV outbreak originating in Wuhan, China: a modelling study. The Lancet. 2020;395(10225):689–697.

2. Baidu. Baidu Qianxi; 2020. Available from: https://qianxi.baidu.com/.

3. Wesolowski A, zu Erbach-Schoenberg E, Tatem AJ, Lourenço C, Viboud C, Charu V, et al. Multinational patterns of seasonal asymmetry in human movement influence infectious disease dynamics. Nature communications. 2017;8(1):1–9.

4. Read JM, Lessler J, Riley S, Wang S, Tan LJ, Kwok KO, et al. Social mixing patterns in rural and urban areas of southern China. Proceedings of the Royal Society B: Biological Sciences. 2014;281(1785).

5. World Health Organization. Disability-adjusted life-year (DALY): Quantifying the Burden of Disease from mortality and morbidity; 2012. Available from: https://www.who.int/healthinfo/global_burden_disease/metrics_daly/en/.

6. WHO Commission on Macroeconomics and Health and World Health Organization. Macroeconomics and health : investing in health for economic development; 2001. Available from: https://apps.who.int/iris/handle/10665/42463.

7. Wikipedia. List of Chinese administrative divisions by GDP; 2020. Available from: https://en.wikipedia.org/wiki/List_of_Chinese_administrative_divisions_by_GDP#cite_note-data2018-3.

8. World Health Organization. Life expectancy and Healthy life expectancy Data by country; 2020. Available from: http://apps.who.int/gho/data/view.main.SDG2016LEXREGv?lang=en.

9. National Bureau of Statistics of China. National Data; 2020. Available from: http://data.stats.gov.cn/english/index.htm.

10. Du L, Wang J, Luo B, et al. Research on disease burden of SARS patients in Guangzhou city. Chinese Journal of Publich Health - Shenyang. 2007;23(3):0379.

11. China says its economy shrank by 6.8% in the first quarter as the country battled coronavirus; 2020. Available from: https://www.cnbc.com/2020/04/17/china-economy-beijing-contracted-in-q1-2020-gdp-amid-coronavirus.html.

12. Ma Z. Q1 Wuhan GDP grows 8.4%; 2020. Available from: http://hb.china.com.cn/2019-04/23/content_40728997.htm.

13. Hubei Government. Hubei Provincial COVID-19 Prevention and Control Headquarters Notice(in Chinese); 2020. Available from: http://www.shanghai.gov.cn/nw2/nw2314/nw2315/nw43978/u21aw1426004.html.

14. Sina News. Summary of school reopening dates (in Chinese); 2020. Available from: https://k.sina.com.cn/article_2345597047_8bcef87702000vctj.html?from=edu.

15. Shanghai Government. Return to Work Guide (in Chinese); 2020. Available from: http://www.shanghai.gov.cn/nw2/nw2314/nw2315/nw43978/u21aw1426004.html.

16. Lee YN. Morgan Stanley says China's first-quarter growth could fall as low as 3.5% due to coronavirus; 2020. Available from: https://www.cnbc.com/2020/02/19/coronavirus-morgan-stanley-economic-forecasts-for-chinas-growth.html.

17. Chinese Center for Disease Control and Prevention. China CDC 2019-nCov case distribution in China; 2020. Available from: http://2019ncov.chinacdc.cn/2019-nCoV/index.html.

18. Lee, Seungjae, et al. Clinical course and molecular viral shedding among asymptomatic and symptomatic patients with SARS-CoV-2 infection in a community treatment center in the Republic of Korea. JAMA internal medicine 180.11 (2020): 1447-1452.

19. Wang, Chia C., et al. Airborne transmission of respiratory viruses. Science 373.6558 (2021): eabd9149.

20. Buonanno, Giorgio, Luca Stabile, and Lidia Morawska. Estimation of airborne viral emission: Quanta emission rate of SARS-CoV-2 for infection risk assessment. Environment international 141 (2020): 105794.

21. Global Times. Hit by coronavirus, Wuhan records 40% GDP contraction in Q1; 2020. Available from: https://www.globaltimes.cn/content/1187207.shtml.
